# Beta oscillations predict the envelope sharpness in a rhythmic beat sequence

**DOI:** 10.1038/s41598-025-86895-y

**Published:** 2025-01-28

**Authors:** Sabine Leske, Tor Endestad, Vegard Volehaugen, Maja D. Foldal, Alejandro O. Blenkmann, Anne-Kristin Solbakk, Anne Danielsen

**Affiliations:** 1 RITMO Centre for Interdisciplinary Studies in Rhythm, Time and Motion, University of Oslo, Forskningsveien 3A, Oslo, 0373 Norway; 2 Department of Musicology, University of Oslo, Oslo, Norway; 3 Department of Neuropsychology, Helgeland Hospital, Mosjøen, Norway; 4 Department of Psychology, University of Oslo, Oslo, Norway; 5 Department of Neurosurgery, Oslo University Hospital, Rikshospitalet, Oslo, Norway

**Keywords:** Human behaviour, Auditory system, Perception, Sensory processing

## Abstract

Periodic sensory inputs entrain oscillatory brain activity, reflecting a neural mechanism that might be fundamental to temporal prediction and perception. Most environmental rhythms and patterns in human behavior, such as walking, dancing, and speech do not, however, display strict isochrony but are instead quasi-periodic. Research has shown that neural tracking of speech is driven by modulations of the amplitude envelope, especially via sharp acoustic edges, which serve as prominent temporal landmarks. In the same vein, research on rhythm processing in music supports the notion that perceptual timing precision varies systematically with the sharpness of acoustic onset edges, conceptualized in the beat bin hypothesis. Increased envelope sharpness induces increased precision in localizing a sound in time. Despite this tight relationship between envelope shape and temporal processing, it is currently unknown how the brain uses predictive information about envelope features to optimize temporal perception. With the current EEG study, we show that the predicted sharpness of the amplitude envelope is encoded by pre-target neural activity in the beta band (15–25 Hz), and has an impact on the temporal perception of target sounds. We used probabilistic sound cues in a timing judgment task to inform participants about the sharpness of the amplitude envelope of an upcoming target sound embedded in a beat sequence. The predictive information about the envelope shape modulated task performance and pre-target beta power. Interestingly, these conditional beta-power modulations correlated positively with behavioral performance in the timing judgment task and with perceptual temporal precision in a click-alignment task. This study provides new insight into the neural processes underlying prediction of the sharpness of the amplitude envelope during beat perception, which modulate the temporal perception of sounds. This finding could reflect a process that is involved in temporal prediction, exerting top-down control on neural entrainment via the prediction of acoustic edges in the auditory stream.

**Supplementary Information:**

The online version contains supplementary material available at 10.1038/s41598-025-86895-y.

## Introduction

Humans, among some other species, show a strong tendency to synchronize their movements to periodic events that express a regular pulse or beat^1^, and their oscillatory brain activity synchronizes with rhythmic sounds^2^. This has been referred to as neural entrainment or neural tracking, and there is an active debate as to whether it is instrumental to temporal prediction and under active top-down control^3–5^.

Neural tracking and entrainment is usually observed as a phase alignment of oscillations in the low frequency range (i.e., delta: 1–3 Hz or theta: 4–7 Hz). However, also power modulations in high frequency oscillations, especially in the beta band (15–25 Hz), have been reported to track the beat in isochronous rhythms and to underlie temporal prediction^1,5–10^. Morillon et al.^8,11^ showed that rhythmic movement enhances acoustic temporal perception in isochronous rhythms. Beta (~ 20 Hz) activity originating in the sensorimotor cortex was time-locked to the expected auditory targets and directed toward auditory brain regions while being coupled to the phase of delta oscillations (1–3 Hz) at the beat rate of the stimuli, possibly reflecting a covert form of active sensing^8^. Moreover, Fujioka et al.^6,7^ reported that beta activity in interacting motor and auditory regions in humans predicts the timing of an isochronous auditory beat already before its onset. During the presentation of isochronous sequences, beta power decreased following sound onset and increased before the onset of the next sound, depending on the tempo. Beta activity might thus represent the internalization of predictable intervals of a beat and reflect the efference copy signal from the motor system generated at the tempo of the beat^1,6,7^.

Most environmental rhythms in our environment, such as music or speech are, however, rather quasi-periodic^3,12^. Nevertheless, neural tracking of the amplitude envelope (changes in the amplitude of a sound over time) of, for example, speech has been shown to support comprehension^4,13–16^. Research supports the conclusion that the sharpness of the amplitude envelope of the speech signal works as an acoustic landmark or “edge” and is a critical factor in driving neural tracking or entrainment, improving speech intelligibility^17,18^. Envelope sharpness can be calculated as the positive first derivative of the amplitude envelope^17,19^. and has been defined as rapid changes in the amplitude envelope that correspond to acoustic onset edges^19^ that have a short rise time, also referred to as a sharp attack^20^. We will use the term attack when referring to rise time throughout the paper.

Importantly, research into human perception of musical rhythm has demonstrated that the perceptual timing precision of a sound depends on the acoustic envelope shape^21,22^. For example, a short sound with a sharp attack is perceived with high temporal precision, whereas a long sound with a gradual (i.e., smooth) attack, like a violin note, gives rise to a perceived pulse that allows for some rhythmic flexibility and features lower temporal precision^22^. In music cognition studies, this has been referred to as the beat bin^22,23^, which denotes the concept that the perceived beat is not defined as a definite point in time but rather by a probability distribution. Hence, the beat bin reflects rhythmic tolerance or the temporal window within which sonic events can occur and still be perceived as (part of) the beat^22–24^. The temporal precision of beat perception seems to vary systematically with amplitude envelope features such as attack and duration^22^, with increasing sharpness of the attack and decreasing duration inducing increased perceived beat precision. We will use the term perceptual timing precision when referring to the width of the beat bin throughout the rest of this paper.

Previous research thus points to a tight link between the sharpness of the amplitude envelope and the precision of beat synchronization. Nevertheless, systematic investigations of the effect of the amplitude envelope sharpness on neural tracking and its perceptual consequences are largely missing (but see^17,25^). Exceptions include studies investigating neural tracking of the amplitude envelope during speech perception^15,19,26^ and a study showing that auditory-motor synchronization can be modulated by acoustic features in a subgroup of participants^27^. The goal of the current study was to reveal neural mechanisms underlying predictions about the envelope shape of sounds embedded in a beat, thereby indirectly addressing how the brain prepares for different levels of perceptual temporal precision.

**Fig. 1:**
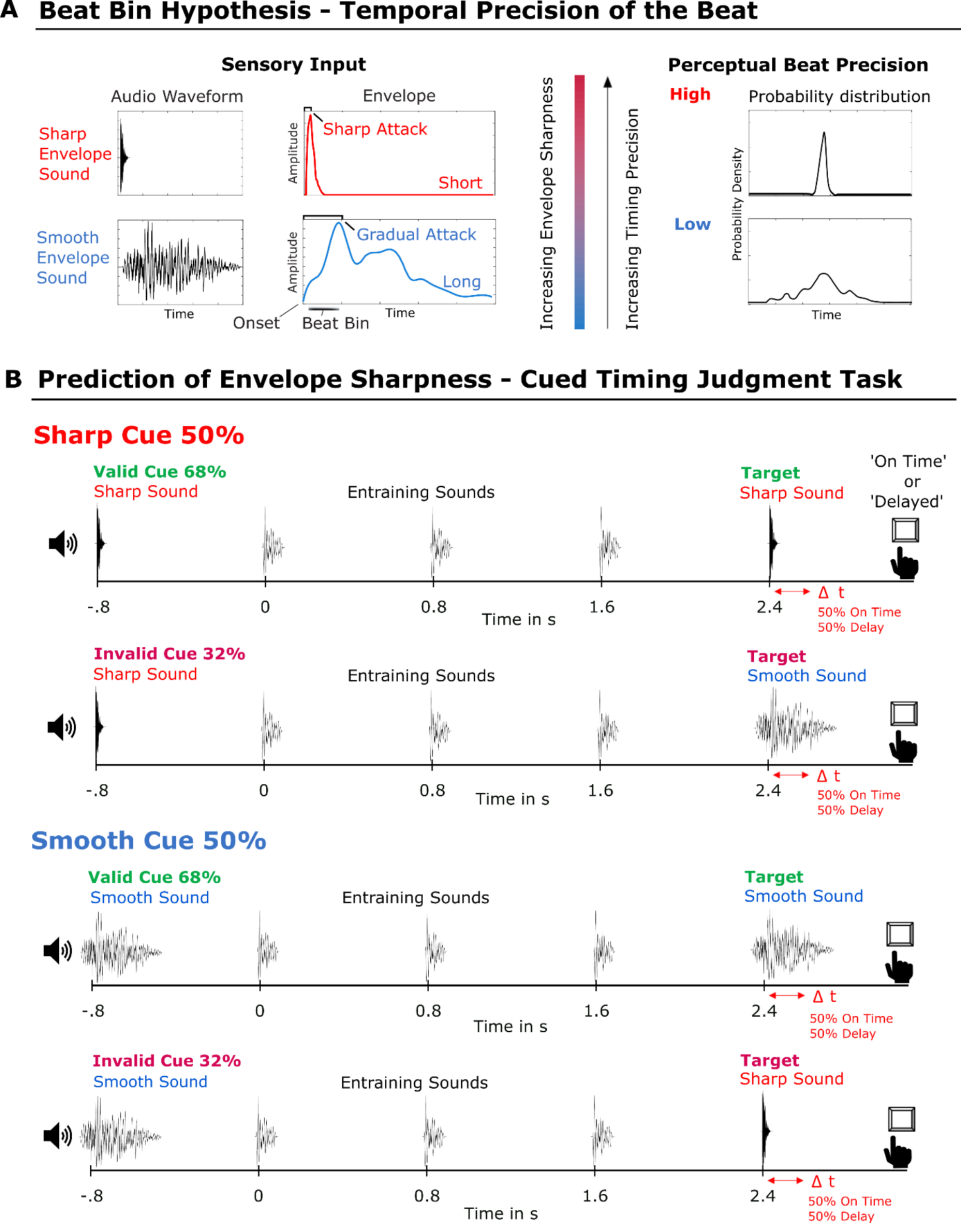
Hypothesis and Experimental Design. (**A**) Exemplary illustration of the beat bin hypothesis. Schematic illustrations of sensory input (Left) and induced perceptual beat precision (Right). The sharpness of the amplitude envelope shape of the sound modulates the temporal precision of the perceived beat. The acoustic shape of the sharp sound (red, short attack and short duration) induces a high-precision beat percept. The acoustic shape of the smooth sound (blue, gradual attack and long duration) induces a low-precision beat percept, which allows for enhanced flexibility with respect to perceived beat timing. The beat bin reflects the temporal width of the beat percept. Schematic probability density distributions for both sound types (Right) resulting from a synchronization task. The probability density distribution represents the variations across instances while participants localize a rhythmic event in time. A smooth envelope sound induces low perceptual timing precision, reflected in a wide and flat probability density distribution, and a sharp envelope sound induces high perceptual timing precision, reflected in a narrow (small standard deviation) probability density distribution. (**B**) Experimental design: two-alternative forced-choice timing judgment task incorporating a sound cue paradigm. Shown are all factor combinations: envelope sharpness, cue validity, and target timing (2 × 2 × 2). Participants were asked to judge whether the target sound was delayed or on time relative to an isochronous sequence of three entraining sounds. Target sounds were on time or delayed with 50/50% probability. The sharp sound cue indicated a sharp target sound, possibly inducing the prediction of high perceptual timing precision (red). The smooth sound cue indicated a smooth target sound, possibly inducing the prediction of low perceptual timing precision (blue). Stimulus plots are a visualization of the waveforms of the sounds used in the study: the sharp sound (red), the smooth sound (blue), and the entraining sound (black). The likelihood of a valid cue was 68%. In the case of an invalid cue (32%), the smooth target sound was presented for the sharp sound cue and vice versa. The slightly earlier onsets of the entraining and smooth sounds schematically illustrate the P-center alignment in the study.

In this study, we examined whether predicting the sharpness of the amplitude envelope affects the temporal perception of sounds. Participants heard a sequence of isochronous sounds and had to judge the relative timing of a target sound at the end of the sequence as either “on time” or “delayed”. We built on the findings of human rhythm-perception research reported above by employing acoustic features that create different degrees of envelope sharpness that have been shown to induce high or low temporal precision in beat perception^23,28,29^. By manipulating the listener’s expectation for the envelope’s sharpness of the target sounds via sound cues, we sought to induce different predictions regarding the perceptual timing precision of a target sound following a beat sequence (Fig. 1). The sound cue was valid in the majority of the trials to enable reliable prediction of the envelope shape of the target.

In accordance with studies investigating temporal prediction and beat perception^1,5–8,30–32^, we hypothesized that cue validity would affect performance in the timing judgment task^33–36^. For sharp target sounds, we expected an increased behavioral performance for the valid (sharp) cue compared to the invalid (smooth) cue, given that these targets require high perceptual timing precision. For the validity effect on the smooth target sounds, our hypothesis was less clear, since there is sparse research into how timing-judgments of smooth envelope sounds are affected by expectations regarding temporal precision. We anticipated enhanced pre-target beta power when participants expected a sharp sound (relative to a smooth sound), supporting predictions of high perceptual timing precision. This might reflect a neural mechanism subserving temporal prediction. As an additional control, and to rule out the possibility that neural responses are purely bottom-up and stimulus-driven, we investigated predictions based on the fluctuations of cue reliability, resulting from the recent history of valid and invalid trials.

## Methods

### Participants

Participants were recruited at the University of Oslo. In total, 28 healthy volunteers participated in the study after giving written informed consent. They were compensated with gift cards worth 300 NOK. Beforehand, the Department of Psychology’s internal research ethics committee had approved the study, which was conducted in agreement with the Declaration of Helsinki. Nine participants were excluded because their performance on the timing judgment task was not significantly higher than chance level (see “Behavioral Analysis” below). The final sample included 19 subjects (13 female, all right-handed, mean age = 25.79 years, std = 3.49 years). All reported normal vision and hearing, no central nervous system damage or disorder, and no cognitive difficulties. They also reported that they did not receive any psychiatric treatment or medication for illness at the time of participation.

### Experimental task and stimuli

Using a cueing paradigm, we manipulated the prediction of the acoustic envelope shape (sharp vs. smooth) of the target sound. Both the cue and the target sound were embedded in a sound sequence that was perceptually isochronous (Fig. 1). The cue was either a sharp (50%) or smooth (50%) envelope sound (further described below), presented at the beginning of each trial. The sound cue indicated the envelope shape of the target sound and participants were told that the cue is informative with respect to the identity of the target sound (sharp or smooth). In the case of a valid cue (68% of trials), the target sound was identical to the cue, whereas in the invalid condition, it was not identical (32% of trials). The target sounds followed three isochronous entraining sounds that were physically constant across all conditions. Participants had to judge the timing of the target sound, i.e., whether it was on time or delayed, in a two-alternative forced-choice (2AFC) task. The ISI between all three entraining sounds was always 800 ms, resulting in a beat frequency of 1.25 Hz (Fig. 1). The ISIs between the cue or target and the entraining sound were adapted so as to ensure perceptual isochrony via a P-center alignment of the stimuli (explained in detail further below).

To induce high or low perceptual timing precision, we utilized sounds with two different acoustic envelope shapes that induce either a high or a low P-center variability, respectively^22,28^. We refer to these sounds as sharp or smooth sounds. These differently shaped sounds were originally designed to investigate acoustic factors influencing P-center location and variability^22,28^. P-center locations are reported in ms relative to the physical onset of the sound. The noise sound with the highest P-center variability results (group mean std = 28 ms) was selected as a sound inducing low perceptual timing precision and referred to as a smooth sound. This sound had a gradual attack (50 ms rise time), a long duration (400 ms), and a center frequency of 100 Hz^22^. The sound with the lowest P-center variability (group mean P-center std = 2 ms) was selected as a sound inducing high perceptual timing precision and referred to as a sharp sound. This sound had a sharp attack (2 ms rise time), a short duration (40 ms), and a spectral centroid of 2370 Hz^28^. The three entraining sounds following the cue sound had a duration of 100 ms, a rise time of 3 ms, and a center frequency of 100 Hz, and they had been shown to induce a P-center variability of std = 7.78 ms^22^, which is between the sharp and the smooth sounds.

Except for the sharp sound, where we assumed a P-center location of 0 ms based on previous studies^28^, all sound types were aligned to participant-specific P-center locations (see the procedure for the P-center estimation below) to ensure that the sound series were perceived as isochronous. For example, for a participant with a P-center location of 15 ms for the entraining sound, the onset of the sound was adjusted to be 15 ms earlier. This particular case would then result in an ISI of 785 ms between, for example, the sharp sound cue (P-center of 0 ms) and the following entraining sound but would induce a perceived isochrony of 800 ms. Given its P-center location of 0 ms (corresponding to the physical onset of the sound), the sharp target sound occured at approximately 2.4 s for the on-time condition. The target sound was also P-center aligned relative to the entraining sounds, resulting in possible earlier or later target onsets.

Overall, and for each factor combination, 50% of the target sounds were on time and 50% were delayed, resulting in a 2 × 2 × 2 factorial design, with factors envelope sharpness (sharp/smooth) x cue validity (valid/invalid) x target timing (on time/delayed) and eight conditions in total (Fig. 1). The sound cues were presented in a pseudorandom order to prevent repetition in successive trials.

Participants indicated whether the target sound was on time (i.e., isochronous to the preceding entraining sounds) by pressing the left green button of a response box (Cedrus Response Pad, Model RB-740, www.cedrus.com*)* with the index finger of their right hand, or delayed by pressing the right red button with the middle finger of their right hand. Participants were given 1.3 s to respond before the next trial was automatically initiated. The inter-trial intervals (ITI) were uniformly distributed between 1.2 and 2.2 s, with a mean length of 1.7 s. Participants were asked to fixate on a central black cross on a gray background that remained on the screen during the entire experimental block.

The target delay threshold was determined individually for each participant after one initial training block consisting of 100 trials. The training block started with a delay of 90 ms for the sharp target sound and 100 ms for the smooth target sound. After the training block, the threshold was automatically adapted. A one-sided binomial test was used to determine whether the performance (accuracy) was significantly higher than chance level (significantly higher than 50% correct). The target delay was increased if the performance was too low (by 30 ms if the accuracy was below 30%, by 20 ms if the accuracy was below 50%, or by 10 ms if the accuracy was above 50% but not significantly higher than chance level). If the performance was too high (approaching 100% accuracy), then the delay was reduced (by 20 ms if the accuracy was above 90% or by 10 ms if the accuracy was above 80%) to avoid behavioral ceiling effects. Individually adjusted temporal delays were held constant across trials and blocks for the main experimental task. The main experimental task consisted of three blocks of 100 trials each, resulting in 300 trials.

### Estimation of the loudness of stimuli

In a pilot experiment, we asked 10 participants (who did not participate in the main experiment) to judge whether the loudness level of the sharp and smooth sounds was perceptually similar to the entraining sound. Participants were allowed to increase or decrease the loudness level of the sharp or smooth sound until it was perceptually similar to the loudness of the entraining sounds. The mean of the amplitude factors for the sharp and smooth sounds across all 10 participants was used as the new amplitude factor for the main experiment (please see stimuli and scripts in the online repository). The sound loudness was at a comfortable level and was constant across participants and blocks.

### P-center estimation

**Fig. 2:**
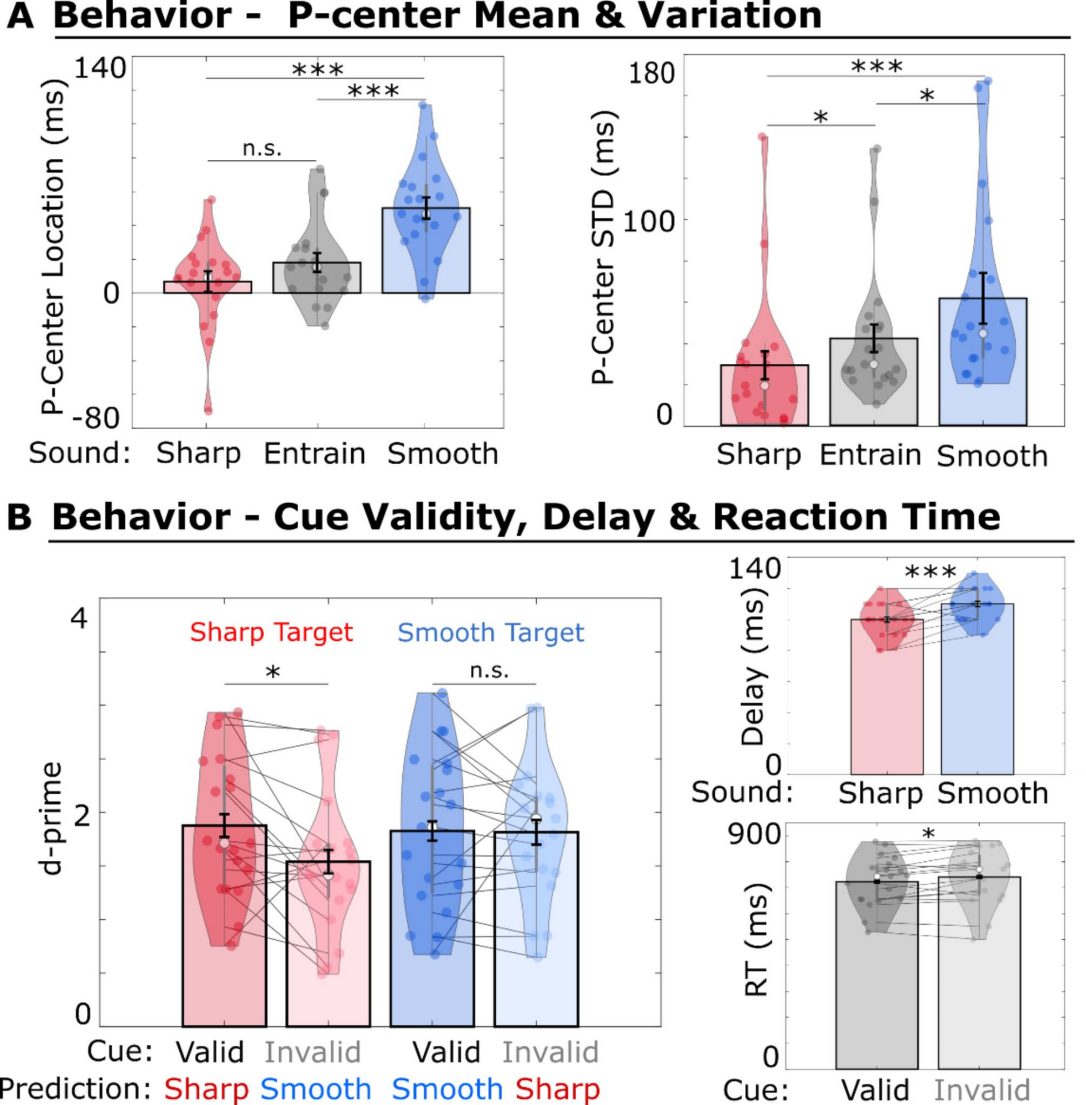
Behavioral Results. Bar plots represent the mean and black error bars represent the adjusted standard error (SE) across participants for a within-subject design, according to the Cousineau-Morey correction^92^. Violin plots show kernel density estimates and data points. Asterisks refer to significant differences between conditions (*< 0.05, **< 0.01, ***< 0.001). (**A**) P-center location and variability (std) for all stimuli, sharp (red), entraining (gray), and smooth (blue) envelope sound from the click-alignment task. (**B**) Behavioral results for the timing judgment task. Left: Valid cue conditions are depicted in dark colors, and Invalid cue conditions are depicted in light colors. Sensitivity (d-prime) was relatively increased for the valid-sharp cue condition (dark red) compared to the invalid-smooth cue condition (light red) (i.e., when participants unexpectedly received a sharp target sound). Sensitivity was not affected by cue validity for the smooth target trials (valid-smooth cue condition [dark blue] versus invalid-sharp cue condition [light blue]). Upper Right: Individual delay threshold results from the training block preceding the main experiment. These thresholds were used for the main experimental blocks. Lower Right: Reaction times for the valid versus invalid cue conditions (pooled over the factor levels envelope sharpness and target timing).

Before the main task, individual P-centers were estimated via a click alignment task (see^22^, for details of the procedure), which is a computerized task to estimate the P-center location and variation (measured in standard deviation). Danielsen et al.^22^ used only four trials for the estimation of the P-center location and variability, since pilot tests showed that this was sufficient to provide a good estimate. For the current study, we increased the number of test trials to get reliable estimates, especially for the smooth sound, since this sound creates the highest perceptual variability in a subject. We used nine trials for the smooth sound, six trials for the entraining sound, and three trials for the sharp sound. We used only three trials for the sharp sound because previous studies determined this to be sufficient^22,28^, and we did not intend to use the result from the click alignment task for the P-center alignment of the experimental stimuli. A previous study^28^ had already shown (using a click-alignment task) that the sharp sound had a grand average P-center location of 0 ms and a standard deviation of 2 ms. Accordingly, we used the respective P-center location for the stimulus alignment in the current study. Please see Fig. 2 and Supplementary Table 1 for the individual P-center locations and standard deviations.

### Post-experiment self-report

All participants completed a self-report (see Supplementary Fig. 1) after finishing the experimental task. The open-ended questionnaire included six open questions regarding their behavioral strategies and their subjectively perceived use of the cue while performing the task.

### Goldsmith musical sophistication index

We collected data for the Goldsmith Musical Sophistication Index (Gold-SMI) questionnaire^37^, which was not used for the current paper (see Supplementary Material).

### Procedure

The session started with participants reading and signing the consent form before we set up the EEG recording and the experimental tasks. Auditory stimuli were played at a comfortable volume from two Genelec speakers (model 8030 W) flanking both sides of the screen.

After performing the click-alignment task to estimate the individual P-center location and variability (approximately 5 min), participants did a training block (lasting approximately 10 min) to estimate the target delay threshold. After a short break, participants performed the three experimental blocks of the main experiment (approximately 12 min each). Participants decided if they would like to take short breaks between blocks. The entire session, including EEG preparation, experimental tasks, breaks, and administration of questionnaires, lasted two to three hours.

### EEG recording

EEG and electro-oculography (EOG) data were recorded using a BioSemi Active Two 64 Ag-AgCl electrode system (BioSemi, Amsterdam, Netherlands). A BioSemi headcap was used to position the 64 electrodes on the scalp of the head, according to the international 10–20 system for electrode placement. Data were sampled at 1024 Hz during online recording. Four external electrodes measured vertical and horizontal eye movements (EOG) and were positioned above and below the right eye, and lateral to the participant’s left and right eyes. Two additional electrodes were positioned on the left and right earlobes for later EEG offline re-referencing.

### Behavioral analysis

We tested whether the performance in the timing judgment task was significantly higher than chance level (more than 50% correct detections) using a one-sided binomial test for the accuracy of the valid sharp cue condition and the valid smooth cue condition.

The binomial test was conducted using the following function: Matthew Nelson (2024). myBinomTest(s, n,p, Sided) (https://www.mathworks.com/matlabcentral/fileexchange/24813-mybinomtest-s-n-p-sided), MATLAB Central File Exchange. If the participant failed to perform significantly better than chance level (*p* > 0.05) in one of the conditions, they were excluded from the analysis. Due to this criterion, nine subjects performing significantly below chance level were excluded, resulting in the final sample of 19 participants.

To quantify the behavioral performance of the timing judgment task, we calculated the d-prime for each condition based on correct and incorrect responses, which is a performance measure for sensitivity based on signal detection theory^38^. We applied a log-linear rule, adding 0.5 to both the number of hits and false alarms and 1 to the number of signal (sum of hits and misses) and noise (sum of correct rejections and false alarms) trials^38^, before calculating the adjusted Hit and False Alarm Rates (FA).

By taking the inverse of the standard normal cumulative distribution function $\Phi ^{{ - 1}}$ (z-score) for the Hit and FA rates, the d-prime (d’) was calculated as follows:

$$d\prime = \Phi ^{{ - 1}} \left( {Hit} \right) - \Phi ^{{ - 1}} \left( {FA} \right)$$

This framework also allows for estimating the response bias (c) separately from sensitivity, which was calculated as follows:

$$c = - \frac{{  \Phi ^{{ - 1}} \left( {Hit} \right) + \Phi ^{{ - 1}} \left( {FA} \right)}}{2}$$

Since we were mainly interested in evaluating whether the cue was behaviorally relevant for the perception of the target and the timing judgment task, we calculated the d-prime and response bias for the factor combinations sharp-valid cue, sharp-invalid cue, smooth-valid cue, and smooth-invalid cue, resulting in a 2 × 2 (envelope sharpness x cue validity) factorial design.

Target reaction times were analyzed only for the correct responses for each of the four conditions, as well as for the valid versus invalid conditions (pooled over the levels of the factors envelope sharpness and target timing).

For the visualization of the behavioral results, the following toolboxes were used: Bechtold, Bastian, 2016. Violin Plots for Matlab, Github Project (https://github.com/bastibe/Violinplot-Matlab, DOI: 10.5281/zenodo.4559847 and https://github.com/anne-urai/Tools/tree/master/plotting/mysigstar.m).

### EEG analysis

#### Data preprocessing

The focus of the analysis of the neurophysiological data was to investigate whether the factor envelope sharpness (sharp versus smooth cue) affected the neural processing of the Entraining sounds and pre-target oscillatory activity. Therefore, we concentrated on the comparison of the sharp versus smooth cue condition, pooling over the two factors target timing and cue validity (both are unknown to the participant before the target arrives), including only trials with correct responses to reduce noise (e.g., attention not on the task, etc.).

EEG data were analyzed offline using custom-written scripts and the Fieldtrip toolbox^39^ in MATLAB (R2020a, Mathworks Inc., Natick, MA, USA). Continuous EEG data were high-pass filtered with a zero-phase Butterworth IIR filter with a half-amplitude cut-off at 0.1 Hz (18dB/oct roll-off) to remove slow drifts in the data and down-sampled to 1000 Hz. The down-sampling was performed via the Fieldtrip toolbox, which in turn makes use of the MATLAB function “decimate,” incorporating a 400 Hz low-pass Chebyshev Type I impulse response (IIR) filter of order 8 that is applied to the data before down-sampling to prevent anti-aliasing. The data were offline referenced to linked earlobes. Noisy segments and bad channels in the continuous EEG data were defined by visual inspection (i.e., muscle artifacts). Following removal of bad channels, an independent component analysis (ICA) was used to identify and remove blinks and horizontal eye movements in the continuous EEG data. Removed ocular components were identified by visual inspection.

The data were referenced to a common average, segmented into epochs of -0.8 to 3.2 s, and time-locked relative to the onset of the first entraining sound. Epochs containing artifacts were removed. Since the different stimuli types were P-center aligned, the alignment to the first entraining sound ensured that trials could be compared across participants. Note that the three entraining sounds, following the cue and preceding the target sound, were presented at a constant rate of 1.25 Hz (SOA 0.8 s) and physically constant across all conditions. Epoched data were demeaned and detrended.

#### Frequency analysis

Time-frequency resolved power was calculated via wavelet transform (Morlet wavelets of 3 cycles) for the entire epoch (-800 to 3200 ms) and the beta frequency range (15–25 Hz) in steps of 1 Hz with a sliding window in steps of 5 ms. Single-trial power values were baseline corrected relative to the power mean across the entire epoch (0–2.4 s) for each frequency bin and expressed in percentage of change. Power values were averaged across frequencies of the beta band (15–25 Hz) to retain beta-power time series. Beta-power time series were smoothed with a moving average of 50 samples (equivalent to 250 ms, using the MATLAB function “smoothdata”) to reduce noise and random fluctuations at high frequencies. This corresponds to applying a low-pass filter, with a stop band at approximately 2 Hz (-3 dB). The average beta-power time series across trials per participant was calculated as input for the statistical analysis. The time window of interest was the interval between the last (third) entraining sound and the target onset, lasting from 1.6 s to approximately 2.4 s.

The onset of the target sound varied across conditions and participants. Depending on the individual P-center locations of the smooth and the entraining sound, the earliest possible target onset was at 2.28 s. (Please see Supplementary Table 1 for individual P-center locations and standard deviations.) We therefore restricted the statistical group analysis to this time window (1.6–2.28 s) to avoid contamination of activity in the pre-target time period via the evoked response to target sounds in some participants.

To reveal slow spectral modulations of beta-power dynamics, single-trial beta-power time series (without prior smoothing) from the time window of interest (last 800 ms before the first possible target: 1.48–2.28 ms) were submitted to a Fourier transform using a Hanning window to calculate the power spectrum for 1–7 Hz (delta and theta band), which was afterward averaged across trials.

In order to identify spectral peaks in the power spectrum indicative of oscillatory activity, the power spectrum of the last 800 ms of the entrainment period before target onset was calculated by applying a Fourier transform to the EEG raw data of the respective time window (1.48–2.28 s) using a Hanning window and subsequently averaging power spectra (1–30 Hz) across trials, task conditions, and participants.

### Modeling cue reliability via transitional probabilities

The rationale behind the calculation of cue reliability is to express the likelihood of observing a sharp or a smooth target sound for the current trial based on the entire history of valid and invalid cue trials, or cue-target transitions.

We used the concept of transitional probabilities (TP) to estimate cue reliability for each trial. To calculate the TP of a sharp cue trial, we divided the number of observed valid cues by the number of valid and invalid cues, to calculate the probability that the current trial is a valid cue-target transition based on the history of trials (observed cue-target transitions). For a sharp cue trial TP, solely observed sharp cue trials (valid and invalid) were considered and for the estimation of the TP of a smooth cue trial, solely observed smooth cue trials were considered. Hence, the cue reliability or TP of a sharp cue trial expresses the probability of receiving a sharp target sound for the current trial, in other words how reliably a sharp cue predicts a sharp target. The complementary TP (invalid divided by valid and invalid) was calculated for smooth cue trials. Accordingly, TP always expresses the probability of receiving a sharp target sound and, in case of smooth cue trials, the probability of an invalid smooth cue to sharp target transition.

Transitional probabilities were modeled with a weighted N-back temporal window for the entire history of trials (1,2,.,k) up to the current trial k^40^.

An exponential decay function was applied to downweight older transitions over newer ones, with a half-life of 4.16 trials (observations), computed as follows:

$$t_{{1/2}} = \tau \ln \left( 2 \right)$$

where $\tau$ is the exponential time constant: $\tau = 6$, giving larger weight to the most recent trials^40^.

This corresponds to a more local integration of observations (leaky integrator). Leaky integrators have been shown to be more biologically plausible in the modeling of brain functions, as opposed to an integration over long time scales with perfect memory^40,41^. A short-term integration (half life of 4) has been argued to represent a more deliberate means of searching for local patterns^40^. Specifically, the cue reliability or TP of a cue-target transition at a trial k was computed as follows:

$$TP_{k} = \frac{{\sum\nolimits_{{n = 1}}^{k} {P_{n} W_{n} } }}{{\sum\nolimits_{{n = 1}}^{k} {P_{n} W_{n} } + \sum\nolimits_{{n = 1}}^{k} {H_{n} W_{{n..}} } }}$$

where W, P, and H are vectors of length k (past trials). W is a weighting vector, with elements:

$$W_{n} = \exp \left( {\frac{{ - n}}{\tau }} \right)$$

for the k-th past stimulus.

If the current trial entails a sharp cue, then:

(1) P elements are defined as 1 for valid sharp cue-target trials (sharp cue to sharp target transitions) and 0 for all other instances (invalid sharp cue to smooth target transitions, valid and invalid smooth cue transitions), and.

(2) H elements are defined as 1 for sharp invalid cue-target trials (sharp cue to smooth target transition) and 0 for all other instances (valid sharp cue to sharp target, smooth cue valid and invalid transitions), for the past trials until k.

If the current trial (k) entails a smooth cue, then:

(1) P elements are defined as 1 for invalid smooth cue-target trials (smooth cue to sharp target transitions) and 0 for all other instances (valid smooth cue to smooth target transitions, valid and invalid sharp cue transitions), and.

(2) H elements are defined as 1 for valid smooth cue-target trials (smooth cue to smooth target transitions) and 0 for all other instances (invalid smooth cue to sharp target, sharp cue valid and invalid transitions), for the past trials until k.

Please note that the weighting vector is applied irrespective of type of cue, corresponding to a temporal weighting.

### Statistical analysis

For the behavioral measures (d-prime and response bias), we conducted two dependent-samples t-tests for valid versus invalid conditions for each target type separately (sharp or smooth target trials). Assumptions of normality were checked via Lilliefors goodness-of-fit test of composite normality and by visually inspecting Q-Q plots. Some distributions deviated substantially from normality. Here nonparametric statistical tests were performed.

Both reaction times for valid versus invalid trials and P-center locations and standard deviations between the three sound types (sharp, smooth and entraining sounds) were compared via nonparametric signed-rank tests.

Statistical group analysis of beta-power time series and spectral modulation of beta-power time series for the two conditions of interest (sharp versus smooth cue conditions) were conducted via nonparametric cluster-based permutation dependent-samples t-tests, correcting for multiple comparisons^42^, applying 10,000 permutations.

Single-trial beta-band power time series were submitted to two-sided independent-samples t-tests contrasting the sharp versus smooth cue conditions to retain individual t-values for the group correlation between neural and behavioral data. Correlations of individual beta-power t-values (sharp versus smooth) with behavioral measures (d-prime and P-center variability) were investigated by performing non-parametric Spearman’s rank correlations using non-parametric cluster-based permutation tests to correct for multiple comparisons, performing 10,000 permutations. Individual d-prime values for the valid sharp envelope cue condition and individual P-center variability results (std) for the smooth sound from the click alignment task were used for this correlation analysis. The correlation analysis was restricted to the pre-target time window of 1.78–2.28 s, according to the results from the statistical group beta-power contrast, showing significant modulation for the factor envelope sharpness (see the “Results” section concerning beta-band power).

To estimate the effect size for cluster-based permutation statistics, we computed average (power or phase) values for each participant and condition across the cluster extent (channels, time bins, and frequency bins, if applicable) and calculated Cohen’s d effect size for within-subjects designs^43^. For correlational analysis, the average Spearman’s rho across the cluster (channels and time bins) is reported as effect size.

## Results

We reasoned that if neural beat tracking of sounds with a varying envelope is under top-down control, then a cue indicating the envelope sharpness of a target sound should affect temporal perception, that is, the judgment of the timing (delayed or on time) of the target sound embedded in a beat. We used a two-alternative forced-choice timing judgment task to test whether cue validity affects detection performance. Participants (*N* = 19) had to detect the timing of a target sound and responded by button press (indicating “delayed” or “on time”). Importantly, the target sound followed three isochronous entraining sounds that were physically constant across conditions and presented at a rate of 1.25 Hz (see Fig. 1).

### Performance in the timing judgment task is affected by the predicted envelope sharpness

To ensure an isochronous beat perception of 1.25 Hz between sounds with different degrees of envelope sharpness, the sounds were aligned according to individual P-center locations measured via a click-alignment task at the beginning of the experiment (see “Methods”). First, we tested whether the stimuli showed the expected perceptual effect with respect to perceptual timing precision, which is reflected by the P-center variability (inverse of precision) measured via the standard deviation (std) across trials (Fig. 2).

We replicated the directionality of the results of Danielsen et al.^22^ and Câmara et al.^28^ showing that the sharp sound had the earliest P-center location relative to sound onset (group mean: 6.84 ms, group std: 27.21 ms) and the smallest variability (group mean std: 29.48 ms, group std of std: 34.12 ms), followed by the entraining sound with an intermediate P-center location (group mean: 18.28 ms, group std: 24.57 ms) and P-center variability (group mean std: 42.77 ms, group std of std: 31.44 ms), and that the smooth sound had the highest mean values for the P-center location (group mean: 50.99 ms, group std: 28.17 ms) and variability (group mean std: 62.41 ms, group std of std: 45.37 ms).

Since we expected the same directionality for the P-center results as reported in Danielsen et al. (2019), we performed one-tailed signed-rank tests for the following comparisons of P-center location, P-center variability and delay thresholds (*N* = 19). These non-parametric tests showed that the two target sound types (sharp and smooth) differed significantly from one another in their P-center location (Z = 3.72, *P* < 0.001, Cohen’s d = 1.63) and variability (Z = 3.24, *P* < 0.001, Cohen’s d = 0.83, Fig. 2). This result is important because it ensured that the acoustic features (attack, duration and center frequency) used for the two cue conditions (sharp versus smooth) had the intended effect on the perceptual timing precision.

The sharp sound did not differ significantly from the entraining sound in P-center location (Z = 1.51, *P* = 0.06), but did differ in P-center variability (Z = 2.15, *P* = 0.016, Cohen’s d = 0.45). The smooth sound differed significantly from the entraining sound in P-center location (Z = 3.81, *P* < 0.001, Cohen’s d = 1.25), and differed significantly in P-center variability (Z = 1.87, *P* = 0.03, Cohen’s d = 0.45). This confirmed that the perceptual effect (P-center location and variability) of the acoustic features of the entraining sound fell between those of the sharp and smooth sounds. The target delay thresholds, estimated from the training block to ensure above-chance-level performance in the timing judgment task, also differed significantly between the sharp (threshold group mean: 100 ms) and smooth sounds (threshold group mean: 110 ms), with a significantly higher mean for the latter (Z = 3.11, *P* < 0.001, Cohen’s d = 1, Fig. 2).

We calculated the d-prime measure to estimate performance and response bias (i.e., the tendency to respond either on time or delayed) in the timing judgment task for the factor cue validity (valid/invalid). Because the target delay thresholds differed between sharp and smooth targets (see paragraph above), the behavioral performance in these two target conditions is not comparable and we therefore performed the validity contrasts separately for the sharp and the smooth target condition.

In line with studies investigating cueing and temporal prediction^33–36^, we hypothesized increased timing judgment performance for the sharp target, if it was preceded by a valid sharp cue compared to an invalid smooth cue. A one-tailed dependent-samples t-test showed that perceptual sensitivity (d-prime) was significantly increased for validly cued sharp targets (valid-sharp cue condition) compared to invalidly cued sharp targets (invalid-smooth cue condition) (t(18) = 2.46, *P* = 0.012, Cohen’s d = 0.56). This indicates that performance in timing judgment deteriorated when the participants expected a target sound with a low envelope sharpness but received a target sound with a high envelope sharpness (Fig. 2). In contrast, there was no effect of cue validity for trials with a smooth target sound, as there was no significant difference between validly cued smooth and invalidly cued smooth targets with respect to the d-prime measures (t(18) = 0.10, *P* = 0.462, two-sided). The response bias showed no significant modulations by validity for the sharp (t(18) = -0.09, *p* = 0.926, two-tailed) or the smooth (t(18) = -1.06, *p* = 0.302, two-tailed) target condition (Supplementary Fig. 3).

Altogether, these results indicate that the cue information about the envelope sharpness of the forthcoming target sound was behaviorally relevant to the temporal perception of the sharp target (Fig. 2).

Based on the well known effect that information about upcoming stimuli improves perception and reduces reaction times^33,36^, we expected a decrease in reaction time for the valid cue condition relative to the invalid one. Non-parametric one-tailed signed-rank tests of cue validity on reaction time did not achieve significance when performed separately for the two (sharp/smooth) target conditions. However, when trials were pooled over both target conditions, cue validity had a significant effect on reaction time (one-tailed signed-rank tests, *N* = 19, z-value = -2.13, p-value = 0.033, Cohen’s d = -0.55).

Interestingly, in the post-experiment self-report, most of the participants (79%) stated that they did not consciously use the information in the cue to solve the task. Participants were instructed not to move during the experiment; 47% reported that they did not move to the beat at all, 37% reported that they moved to the beat on some occasions, and 16% reported that they moved to the beat during the experiment. The majority (68%) of the participants indicated that they had the impression that it was easier to judge the timing of the sharp target sound. See “Supplementary Material” for the other results of the questionnaire.

### Beta-band power is modulated by the predicted envelope sharpness

To reveal the neurophysiological processes underlying the prediction of envelope sharpness, we focused on pre-target beta oscillations during the entrainment interval, aligned to the first of the three entraining sounds. We concentrated on the main factor of envelope sharpness, comparing trials starting with a sharp sound cue to trials starting with a smooth sound cue.

Grand average spectra across participants were calculated for the entire entrainment period (0–2.4 s). The spectra revealed narrowband peaks of power in the theta (4–6 Hz), alpha (8–12 Hz), and beta (15–25 Hz) frequency ranges, indicating the involvement of oscillatory activity (Supplementary Fig. 2).

**Fig. 3:**
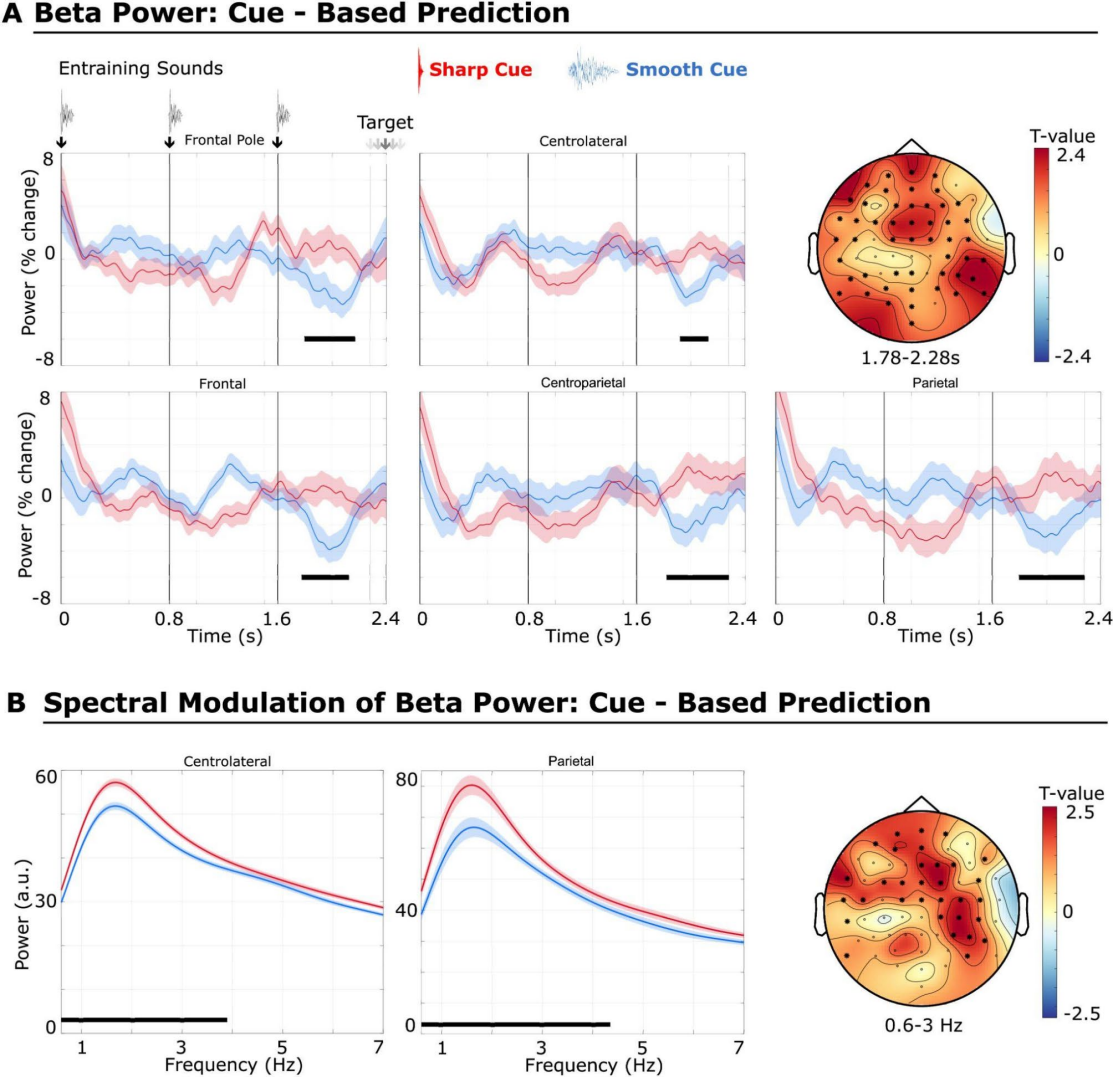
Beta power effects for the sharp versus smooth target prediction based on the cue. Shaded areas represent the adjusted standard error (SE) for a within-subject design, according to the Cousineau-Morey correction^92^. Black lines indicate significant differences in the temporal dimension. The spatial extent of the significant clusters is depicted by bold stars in the topographical plots. The small gray arrow symbols for the target depict multiple possible onsets for the target according to individual P-Centers. (**A**) Pre-target beta-power time series (percentage change) for sharp (red) versus smooth (blue) cue condition. Pre-target beta power is significantly enhanced for the sharp cue condition. For visualization purposes, beta power was averaged over significant sensors of selected electrode placement sites: Frontal Pole (Fpz, Fp1, Fp2), Frontal (Fz, F1, F2, F4–7), Centrolateral (C4–6, T7), Centroparietal (CP4, CP6, TP7, TP8), Parietal (Pz, P1, P4–8). (**B**) Spectral modulation (Fourier transform) of beta-power time series for the sharp (red) versus smooth (blue) cue condition. The spectral modulation of the pre-target beta-power time series is significantly enhanced for the sharp cue condition within the delta (1–3 Hz; topographical plot shown on the right) and theta (4–7 Hz) frequency ranges (data not shown). The spectral modulation of beta power was averaged over significant sensors of selected electrode placement sites: Centrolateral (C4–6, T7), Parietal (P4, P6, P8).

The smoothed beta-power time series revealed an amplitude modulation at the rate of the entraining sounds for the sharp cue condition at certain electrodes. For example, beta-band power at left (C5, T7) and right (C4, C6) centrolateral sensors evinced an Event-Related Depression (ERD) following the entraining sound and a beta-power increase (Event-Related Synchronization, ERS) shortly before the next entraining sound (Fig. 3). Beta-power time series showed different dynamics depending on the cue that were already evident after the first entraining sound and throughout different time segments of the entire entrainment period (Fig. 3).

For the following analysis, we concentrated on the time period shortly before target onset, starting with the last (third) entraining sound and ending with the first possible target sound onset (see “Methods” for details). This corresponds to the time period 1.6–2.28 s relative to the first entraining sound. Based on previous studies investigating beat perception and temporal processing^6,7,10,30,44^, which reported a positive relationship between beta power and perceptual timing performance or predictive timing, we also expected a beta power increase for the sharp cue condition, which requires enhanced perceptual temporal precision. The statistical contrast (one-tailed cluster-based permutation test, *N* = 19) between sharp versus smooth cue conditions revealed that beta power was significantly enhanced for the cue condition predicting a sharp target sound (cluster T-sum = 4482.19, cluster P-value = 0.026). This relative increase in beta power was most pronounced approximately 500 ms prior to the target sound until the earliest possible occurrence of that target sound (range = 1.775–2.28 s), at frontal, central, temporal, parietal, and occipital sites. The effect size for the average power values across channels and time points was Cohen’s d = 0.83.

In order to test if smoothing beta power time-series (smoothing corresponded approximately to a 2 Hz low-pass filter, see Methods) affects the results, the statistical analysis was repeated without smoothing, which yielded similar results, with a significant increase in pre-target beta power for the sharp cue condition (*N* = 19, cluster T-sum = 2301.65, cluster P-value = 0.007, one-tailed, Cohen’s d = 1.31, see Supplementary Fig. 4). To control for evoked responses and possible confounding effects on pre-target beta power, we subtracted the event-related potential (ERP, phase-locked) from each single trial waveform to retain induced (non-phase-locked) beta power responses^7,9,10,44,45^. The cue-based prediction results were unaffected, showing a significant increase in beta power for the sharp envelope cue condition (cluster T-sum = 4543.60, cluster P-value *p* = 0.026, Cohens d = 0.82, see Supplementary Fig. 5).

In order to test if the effect is constrained to the beta band, we performed the same analysis on the alpha band, which seemed to indicate a comodulation of alpha and beta power time series (Supplementary Fig. 6), but the cue-based prediction contrast between sharp versus smooth envelope cue was not significant for the alpha band (*N* = 19, Cluster T-sum = 6112.44 *p* = 0.116, one-tailed).

To control for differences in the physical features between the sharp and smooth cues at the beginning of the trial and a possible confounding effect on pre-target beta power (2.4 s later), we also analyzed the dynamics of cue reliability over time.

**Fig. 4:**
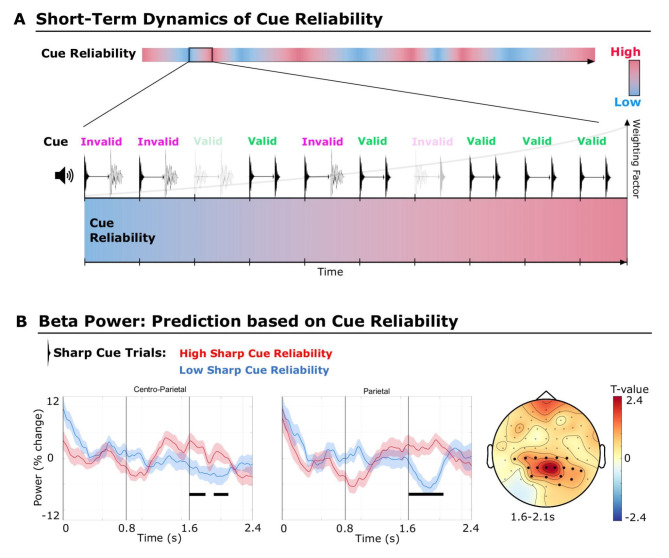
Although the cues were overall valid in 68% of the cases, cue reliability showed fluctuations on a short time scale due to the random nature of the experiment. (**A**) Cue reliability varied dynamically based on the history of recent valid and invalid cue trials. Cue reliability for each cue type (sharp or smooth) was calculated via transitional probabilities (see Methods), mirroring the probability of a valid cue based on the proportion of valid and invalid cue-target transitions for this cue type (visualized via audio waveforms). The weighting factor indicates the exponential decay function, that was used to downweight trials that were longer back in time, i.e., recent trials get a higher weight. (**B**) Pre-target beta-power time series for high (75th percentile or upper quartile) versus low (25th percentile or lower quartile) cue reliability for sharp cue trials, i.e., a high versus low transitional probability to receive a sharp target sound following a sharp cue. Differences are shown for the sharp cue condition only, to control for perceptual confounds caused by physical differences between the sharp and the smooth sound cue. Beta power was modulated in the same direction as for the cue-based contrast, showing increased beta power for a high relative to a low sharp cue reliability. For visualization purposes, beta power was averaged over significant sensors of selected electrode placement sites: Centroparietal (CPz, CP1–3, TP5), Parietal (Pz, P1 -4, P6).

Overall the cue validity of an experimental block (several minutes) is 68%, but given the random nature of the experiment, fluctuations in the probability of the cue to be valid are to be expected within short time windows (several seconds). For some short periods, the number of successive valid cue trials will increase and therefore the confidence in the cue (cue reliability) increases for the time periods. We used transitional probabilities, here reflecting valid and invalid cue-target transitions, to calculate the dynamics of cue reliability on a short time scale (see Fig. 4A). It has been shown that the brain can track random fluctuations of transitional probabilities^40,46,47^. Therefore, we hypothesized that, if there is a top-down mechanism predicting the envelope sharpness for a specific target, differences in cue reliability should modulate beta-power oscillations in the same direction as different cue conditions. In other words, if for a short period sharp cues are reliable (valid), that is, followed by sharp targets, pre-target beta power should increase in the following trials. However, if sharp cues are mostly unreliable and followed by smooth targets, pre-target beta power should decrease.

In practical terms, we modeled cue reliability via the probability of receiving the correct target sound for each trial by taking into account valid and invalid cues of previous trials (for details, see “Methods” and Fig. 4A). This allowed us to contrast the high (75th or upper quartile) versus low (25th or lower quartile) cue reliability trials for each cue condition (sharp and smooth) separately. It is important to note that these statistical contrasts entail no physical stimulus differences for the cue, but are based on dynamic cue confidence. The results showed that beta-power time series were modulated in the same direction (Fig. 4B), with significantly increased pre-target beta power for high (75–100%) sharp cue reliability trials (i.e., high transition probability (TP) to receive a sharp target), as opposed to low (1–25%) sharp cue reliability trials (i.e., low TP to receive a sharp target sound). This effect was apparent for the sharp cue condition (cluster-based permutation statistics, *n* = 19, cluster T-sum = 1894.01, cluster P-value = 0.016, two-tailed) and was most pronounced between 1.60 and 2.095 s for parietal sensors. The effect size for the average power across channels and time was Cohen’s d = 1.47. For the smooth cue condition, the high versus low TP contrast was not significant.

Beta-power time series preceding the target by approximately 800 ms (1.48–2.28 s, to avoid evoked responses by early target onsets in some participants; see “Methods”) were submitted to a spectral decomposition to investigate frequency modulations of beta-power dynamics.

The power spectra of the beta-power time series showed a peak at approximately 1.6 Hz for both conditions, revealing that the strongest modulation of the beta-power time series was in the delta range (1–3 Hz), entailing the beat rate (1.25 Hz) of the entraining sound (Fig. 3B). Beta-power dynamics showed a significantly stronger modulation at the delta frequency range (Fig. 3B) for the sharp cue condition ( cluster-corrected permutation test, *n* = 19, cluster T-sum = 2019.12, cluster P-value = 0.019, two-tailed). This modulation was also apparent for one electrode (AF7) for the theta (4–7 Hz) frequency band. These frequency modulations were seen at central, frontal, and parietal sites (Fig. 3B). The effect size for the average power across channels and frequency bins was Cohen’s d = 0.99.

### Beta-band power correlates with performance in the timing judgment task

We tested neural-behavioral correlations to reveal the behavioral relevance of the identified pre-target beta-power modulation effects (i.e., the enhancement of beta power for the prediction of a sharp sound), inducing high perceptual timing precision.

**Fig. 5:**
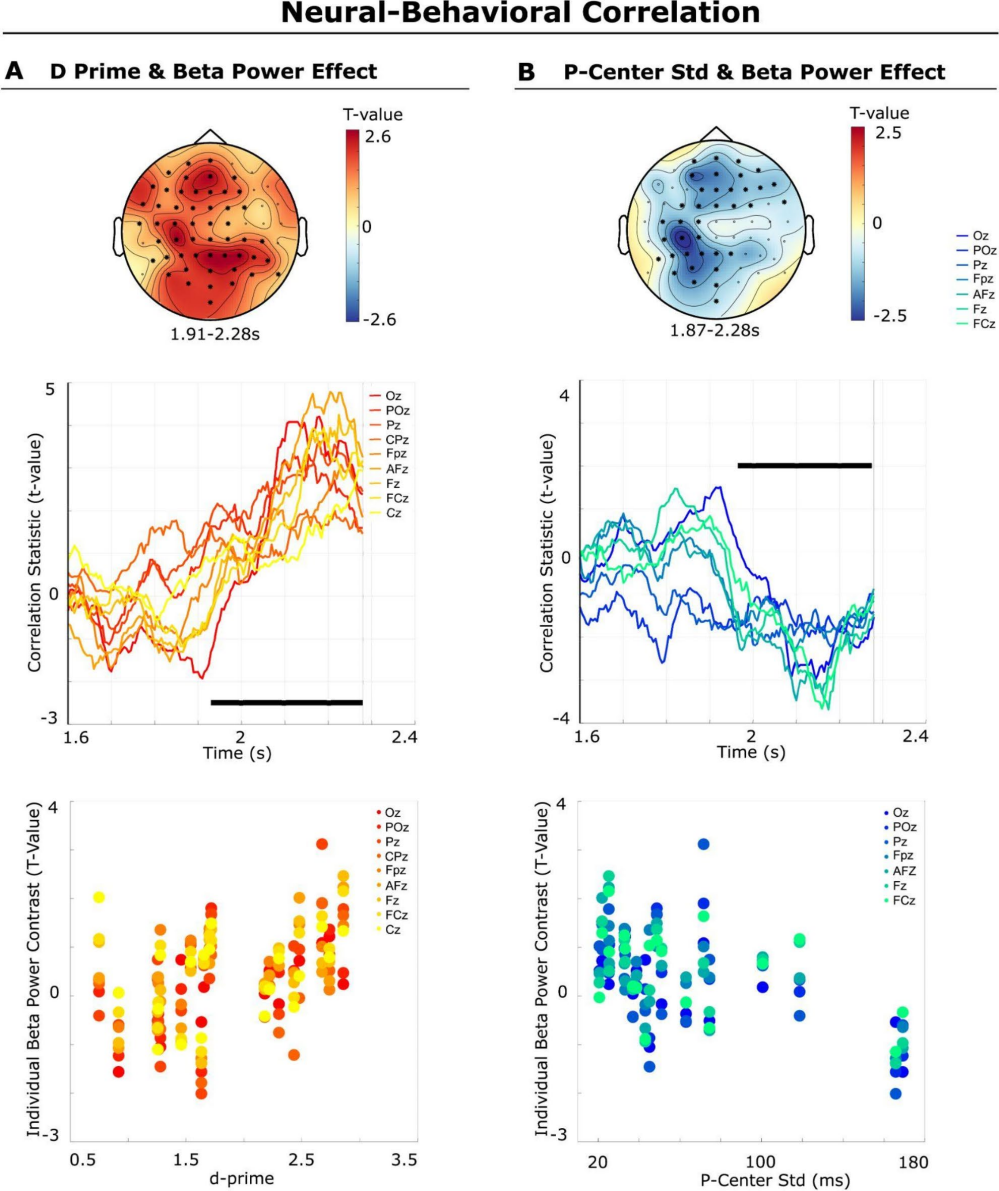
Neural-Behavioral Correlation. Top: topographical distribution of the correlation effects. Middle: correlation time courses of the significant midline sensors. Bottom: correlation at the individual subject level (each dot represents a participant for the significant midline sensors). (**A**) Correlation between the behavioral d-prime measure for the valid sharp cue condition and the individual beta-power contrast (t-value) between the sharp versus smooth cue conditions. There was a positive correlation between the individual beta-power effect and the d-prime result for the timing judgment task. (**B**) Negative correlation between the behavioral P-center variability measure (std) for the smooth sound and the individual beta-power contrast (t-values). Together, these results support the hypothesis that pre-target oscillatory beta activity encodes the predicted envelope sharpness and temporal precision of the upcoming target sound.

As our initial behavioral index, we selected the sensitivity measure (d-prime) that reflects the performance in the timing judgment task in the valid sharp cue condition and investigated its relation to the individual beta-power t-values (sharp versus smooth cue condition) via Spearman correlations. Based on previous studies showing that beta power correlates positively with temporal accuracy and prediction^1,6,8,30,48,49^, we had a clear hypothesis about the directionality of the effect: a positive correlation between timing judgment performance and beta-power t-values. The group-level test showed a significant correlation (cluster t-sum = 5041.77, cluster P-value = 0.008, one-tailed) between the d-prime (for the valid sharp cue condition) and the beta-power t-contrast. This correlation was driven by an effect starting at 1.905 s at the earliest and lasting at the most up to 2.28 s (i.e., shortly before target onset), with an extended topography over frontal, temporal, parietal, and occipital sites (see Fig. 5). The average effect size across the cluster (channels and time bins) was rho = 0.54. To ensure that the correlation analysis was not confounded by individual differences in task difficulty, due to the individually adapted thresholds, we repeated the analysis by correlating individual thresholds for the sharp target (instead of the d-prime of the valid sharp cue condition) with individual beta-power t-contrast values. This correlation was not significant, indicating that the correlation result was likely not confounded by variability in difficulty level (Supplementary Fig. 7).

As a second behavioral measure, we were interested in the P-center variability, which increases with decreasing precision in the perception of the temporal location of a sound^22^. Since other studies have shown a positive relationship of beta power with temporal prediction and accuracy^1,6,8,30,48,49^, we hypothesized a negative correlation between P-center variability (inverse of precision) and the individual beta-band power t-contrast. Non-parametric cluster-based permutation tests on the group level revealed significant negative correlations (cluster t-sum = -2478.82, cluster P-value = 0.049, one-tailed) between the individual P-center variability (standard deviation of the smooth sound) and individual beta power t-values. The correlation was most pronounced between 1.87 s and 2.28 s, with a topographic distribution encompassing the frontal, central, and posterior parts of the sensor space (Fig. 5). The average effect size across the cluster (channels, time, and frequency bins) was Spearman’s rho = -0.52. The most pronounced effects were found at posterior sites, followed by anterior sites.

These results indicate that the participants who showed the strongest beta power modulation by the cue (i.e., larger beta-band power t-value for the sharp cue condition) both performed better on the timing judgment task (positive correlation) and showed a higher perceptual timing precision when estimating the P-center of the smooth sound (i.e., lower P-center variability; negative correlation).

## Discussion

Music and speech are structured in time by rhythmic modulations in sound intensity. In speech, slow temporal modulations reflect the syllabic (~ 5 Hz) and phrasal (0.5–2 Hz) rate^13,50–52^. In music, slow modulations (0.5–3 Hz) reflect acoustic fluctuations near the beat rate^13^, underlying the perception of beat, meter, and grouping^13,53–55^. In the current study, we investigated how predictive information about low-level features of acoustic stimuli, namely, the sharpness of the envelope, serves the prediction of the timing of a target that is embedded in such a musical beat. We argue that the acoustic envelope shape is closely related to perceptual timing precision^22,23^ and we show that neural beta activity encodes the prediction of envelope sharpness.

Flexibility in perceptual timing precision allows us to perceive regularity in acoustic information that does not show a strictly periodic pattern or has a shape that renders its perceived temporal position indistinct, as can be the case in language^15,56^ and music^21,22^. Although neural tracking is often demonstrated for strictly isochronous stimuli, the neural mechanisms seem to tolerate a certain amount of temporal variability^4^. Such tolerance for temporal variability is also more adaptive, given that most environmental events do not follow strict isochrony^56^ and tend to vary in their acoustic envelope shape. Deficits in the detection of the attack and impaired audio-motor synchronization to a beat are apparent in developmental dyslexia^57,58^. This provides additional evidence that the sharpness of the speech envelope is critical to rhythm synchronization and language comprehension^51,57–62^.

Likewise, research on the perception of timbre and source identity has shown that when attack information is removed from the sound, the perceiver’s ability to identify the instrument decreases drastically^63–66^. These results suggest that acoustic edges, and in particular the shape of the attack, carry important information about timbre and source identity in music perception.

### The expected sharpness of the acoustic envelope modulates temporal perception

We replicated the results of previous studies^22,28^, as the sound with a sharp acoustic envelope had a significantly lower perceptual variability (i.e., a higher perceptual timing precision) than did the smooth sound. These results verify that the target sounds affected perceptual timing precision in the intended direction.

Perceptual sensitivity was affected by the cue, supplying clear evidence that the sharpness of the acoustic envelope indicated by the cue was behaviorally relevant and had an impact on the temporal perception of the target.

Timing judgment performance was modulated by cue validity only for target sounds with a sharp envelope. This means that expecting a target with a smooth envelope was detrimental to the judgment of the timing when the actual target was a sharp envelope sound that required high perceptual timing precision. In contrast, cue validity did not significantly affect behavioral performance for target sounds with a smooth envelope, which induce low perceptual timing precision. This could reflect a ceiling effect, since judgment of the timing of the smooth target requires a lower temporal precision than the sharp target. A cue that erroneously signaled high temporal precision (i.e., an invalid cue for a sharp target), was not detrimental to task performance.

The performance enhancement for the sharp target condition by the valid sharp cue is a pivotal finding because it shows that the top-down prediction of the sharpness of the envelope affects temporal processing. This indicates that the neural prediction of the envelope shape also incorporates a prediction of the perceptual timing precision of the target sound.

Interestingly, the post-experiment self-report (an open-ended questionnaire) contradicted the behavioral and neurophysiological results. Most of the participants (79%) stated that they did not use the cue to solve the timing judgment task. This suggests that most participants were not consciously aware that the perceptual system uses the cue information to predict the acoustic shape of the target and, in turn, the requisite perceptual temporal precision for performing the task. On the other hand, most people (68%) reported that it was easier to judge the timing of the sharp target sound than the smooth target sound. This is interesting because it coincides with the behavioral advantage of the cue, which only arose for the sharp target sound. Altogether, our findings show that the neural system exploited the information conveyed by the cue to predict different levels of envelope sharpness and the related perceptual timing precision of sounds in a top-down manner while tracking a beat.

### Beta-band power encodes predictions about the acoustic envelope sharpness and temporal precision

At the neural level, the study replicated previous findings that modulations in beta-band power synchronize with a regular beat^1,7^ and encode temporal predictions^8,31^. Importantly, this study revealed that beta power is modulated in a top-down manner by the cue, enabling the neural system to flexibly adapt to different acoustic envelope shapes in accordance with task-specific demands, thereby enhancing sensory selection and performance.

Overall, the results are in line with studies indicating that temporal sensory predictions are implemented via beta oscillations^5–8,31,49,67^. Relatedly, Arnal et al.^30^ reported that pre-target beta power was enhanced for correctly detected targets in a timing judgment task, along with a significantly enhanced cross-frequency coupling between pre-target delta phase and beta power.

In addition, brain–behavior correlations indicated that the participants who showed a higher beta-power modulation for sharp versus smooth sound cues both performed better on the timing judgment task of the sharp sound (i.e., larger d-prime; positive correlation) and were more precise at estimating the P-center of the smooth sound (i.e., lower P-center variability; negative correlation). This provides important support for the behavioral relevance of neural beta power for predicting beat timing and the temporal precision of sounds in a beat sequence.

Moreover, beta-band activity was modulated most strongly in the delta range (1–3 Hz), which entails the beat rate, i.e. the frequency of the entraining isochronous sounds (1.25 Hz) preceding the target. Importantly, this spectral modulation was relatively enhanced for the sharp compared to the smooth cue condition within the delta (1–3 Hz) frequency ranges during the last entrainment interval before the target. Our results are in line with the co-modulation of delta and beta oscillations carrying temporal predictions found by Morrillon et al.^8^. The authors showed that top-down influences via the motor system sharpen sensory processing by encoding temporal predictions via beta oscillations^8^. Beta oscillations were directed toward sensory regions and coupled to delta oscillations. The authors argued for the central role of beta oscillations in representing temporal information and encoding top-down predictions, realized via a covert form of active sensing with motor areas providing contextual information to sensory regions^8^. Crucially, in the present study, we demonstrate that beta oscillations are more strongly modulated at the beat rate (1–3 Hz) for the sharp envelope cue. This modulation conveys information about the predicted envelope sharpness, and therefore the requisite perceptual timing precision of a sound in a beat sequence. Since we did not perform a cross-frequency coupling analysis as Morillon et al.^8^, we lack direct support for a respective phase reset of delta oscillations caused by beta bursts, and the paradigm does not allow us to investigate phase amplitude coupling. The modulation of the amplitude of beta activity in the delta range could therefore also be caused by other neural mechanisms.

The results of the current study can be interpreted in the light of the active sensing framework, although we can not provide direct support since we did not investigate the neural generators of the effect nor connectivity. Beat perception has been shown to engage the motor cortico-basal ganglia-thalamo-cortical (mCBGT) circuit, with the supplementary motor area (SMA), dorsal striatum, and putamen as important nodes^68^. This is complemented by studies examining single-cell and microcircuit activity in macaques^1^. With the action simulation for auditory prediction hypothesis (ASAP), Cannon and Patel^68^ proposed a causal role for the motor system in beat-based temporal predictions and suggested that the prediction of beat timing is communicated through the dorsal auditory pathway connecting the premotor, parietal, and temporal cortices. In line with this, Fujioka et al.^6^ reported beta modulations in auditory, motor-related brain regions and the precuneus to be involved in predictive beat timing. Since we did not source-localize the beta-power effect in the current study, the interpretation of the scalp topography remains somewhat speculative. But the topography encompassed not only fronto-central areas, but also central and parietal regions of the scalp and was widespread.

One suggested scenario in the active sensing framework is an oscillatory phase reset being implemented by the corollary discharge from the motor command that generates a sensory sampling action, informing the sensory region about the temporal arrival of the sensory input^4^. It has been proposed by Rimmele et al.^5^ and others, that a top-down predictive phase reset of low-frequency (e.g., delta) oscillations (i.e., an anticipatory phase reset) in sensory regions could be implemented via the motor system’s encoding of temporal predictions in beta-band activity^1,4,5,69^. In the predictive coding framework, it also has been proposed that beta oscillations might be involved in top-down predictions^70–72^ and the endogenous activation of neural populations that are relevant for the task^72^. In the current study, beta activity was modulated at the rate of the beat and encoded the expected levels of envelope sharpness up to several hundred milliseconds before target sound onset. However, if this represents a predictive process of the motor system that informs sensory regions remains speculative until the underlying neural sources, connectivity and phase amplitude coupling are investigated in future studies.

To rule out possible confounds caused by different stimulus properties of the sound cues at the start of the sequence, we calculated the likelihood of a sharp target sound for each trial based on the short-term dynamics of cue reliability (transition probabilities), that is, the history of trials. This more dynamic measure allowed us to contrast trials with high versus low probability to receive a sharp target sound within the sharp cue condition. Beta power was still modulated in the same direction, with enhanced pre-target beta power for trials with a high versus a low probability to receive a sharp target sound. This rules out purely bottom-up-driven neural responses for the beta-power results and confirms a top-down contribution to the prediction processes that incorporate the information provided by the cue. The comparison of high versus low cue reliability probably also mimics a manipulation of high versus low cue validity, which was not part of the experimental design, since overall cue validity was 68%.

There is a vast amount of studies showing how alpha power (8–12 Hz) in sensory areas is modulated by expectation and top-down attention in the visual, somatosensory but also auditory domain^73–79^. Therefore, we analyzed if alpha oscillations might also be involved in the cue-based prediction of envelope sharpness. This revealed that alpha power time-series for the sharp and smooth cue condition seemed to show similar dynamics as beta power, but there was no significant pre-target alpha power difference for the cue-based prediction of envelope sharpness, i.e., perceptual temporal precision of the target. It has been argued that alpha oscillations might be involved in spatial and feature-related attention, while beta oscillations might underlie temporal attention^31,80^. Still, a study by Wöstmann et al. has shown that lateralized alpha power synchronizes with the tempo of stimuli, reflecting a spatiotemporal filter subserving auditory attention to speech^81^. However, the experimental task involved a response to certain features of the stimuli, while in our study, participants had to judge the timing of the target. In a related study that involved auditory spatial attention, but also a timing judgment task, we reported significant modulations in laterized beta power and not the alpha band^31^.

Interestingly, a recent study^82^ revealed that the modulation of EEG beta and gamma (> 30 Hz) activity in response to a rhythm was correlated to grammar abilities in children, supporting the idea that neural beat tracking may be an underlying mechanism that is important for both language and music processing. This is in line with studies showing an impairment in the ability to synchronize movements to a beat in children with dyslexia^57^, who also have been shown to have difficulties with detecting the attack (rise time, e.g. sharp onsets) of sounds^51,60^.

Envelope sharpness has been reported to modulate speech tracking^17,19^ and the precision of beat synchronization, which has been shown in former studies^22,23^ and this was replicated with the behavioral data in the current study. Therefore, we believe that the neurophysiological results of our study - the encoding of the expected envelope sharpness in pre-stimulus beta power - could represent a common predictive mechanism that supports neural tracking of- and entrainment to syllables in speech and beats in music.

## Limitations and future research

Fujioka et al.^6^ source-localized beta activity to the auditory cortex and found that the beta-event-related synchronization (ERS, power increase shortly before the sound onset) represented the predicted timing of the next sound in a beat sequence. We did not perform a source localization of the beta activity (i.e., the analysis of the ERS and event-related desynchronization [ERD]) in relation to the entraining sounds, as this was beyond the scope of this paper.

If the listener expected a sharp target sound, then beta-power time series were comodulated with the entraining sounds for some electrodes, with a power increase before the next entraining sounds and a power decrease after the sound, resembling the ERS and ERD described by Fujioka et al.^6,7^.

However, for the condition inducing a prediction of a smooth target sound, the modulation of the beta activity was quite different, with a beta-power peak halfway between the first and second, and second and third, entraining sounds (around 0.4 s and 1.2 s) for several anterior sensors. This is a highly interesting observation and should be further investigated in a future study conducting EEG source analysis.

Studies investigating rhythm-based and cue-based temporal expectations have shown functional dissociations in behavior and provided evidence that both processes might rely on distinct brain circuits^35,83,84^. In the current study, we did not intend to dissociate cue-based and rhythm-based temporal predictions. Accordingly, the task design does not allow for an investigation of a possible dissociation between these cognitive mechanisms. Our goal was to modulate the beat-based neural tracking process in a top-down fashion via a cue. Still, multiple distinct mechanisms might have been at play and acted in synergy, creating the behavioral benefit of the cue in a task that involves rhythmic expectations.

Approximately one third (9 out of 28) of the participants did not perform significantly better than chance level in the timing judgment task, indicating they were not able to do the task and were therefore excluded from further analysis. A possible reason for this high percentage is that the threshold adaptation method used (see Methods) might not have been optimal. An adaptive threshold estimation method^78,85^ or staircase-like procedures^86^ might have improved the estimation of the target delay thresholds.

Another limitation of the study might be that targets differed not only in P-center variability but also in spectral features (center frequency). Danielsen et al. (2019) systematically investigated the influence of all three acoustic features on the P-center variability and found that only attack and duration had a significant influence on the variability of the P-center (here referred to as perceptual timing precision) and not frequency. We therefore believe that the prediction of the attack and duration, i.e., acoustic features that determine the envelope sharpness of sounds, were contributing to the temporal perception effects found in the current study. To rule out spectral features as a possible confounding factor, a future study could control for the center frequency of the stimuli and only change the shape of the attack of the sounds in order to manipulate P-center variability.

## Conclusions

Taken together, our findings show that the brain leverages prior information about the sharpness of the acoustic envelope to predict different levels of perceptual timing precision of future sounds in a rhythmic sequence of events, and that this process is at least partly under top-down control. Importantly, the results reveal that beta activity could be involved in the neural tracking of not only strictly isochronous events but also quasi-periodic patterns, which predominate in speech and many musical styles^87–91^.

The pre-stimulus modulation in beta oscillations possibly reflects a predictive mechanism that dynamically adjusts neural entrainment to expected acoustic landmarks in music or speech.

## Electronic supplementary material

Below is the link to the electronic supplementary material.

Supplementary Material 1

## Data Availability

The experimental data, stimulus presentation and custom analysis code that support the findings of this study are openly available at: https://osf.io/rb3ap/.
