# Supplementary material for: Beta oscillations predict the envelope sharpness in a rhythmic beat sequence
Source: Sci Rep. 2025 Jan 28;15:3510. doi: 10.1038/s41598-025-86895-y (PMC11775266; doi:10.1038/s41598-025-86895-y)
Supplement: Supplementary file 1 — Supplementary Material 1 [file 41598_2025_86895_MOESM1_ESM.docx]

**Supplementary Material for:**

**Beta Oscillations Predict the Envelope Sharpness in a Rhythmic Beat Sequence**

Sabine Leske, Tor Endestad, Vegard Volehaugen, Maja D. Foldal, Alejandro O. Blenkmann, Anne-Kristin Solbakk, Anne Danielsen

**Goldsmith Musical Sophistication Index**

We included the Goldsmith Musical Sophistication Index (Gold-SMI) ^1^, a self-report inventory indicating the level of musical experience. This score incorporates aspects from five sub-scales measuring self-reported active musical engagement, perceptual abilities, musical training, singing abilities, and sophisticated emotional engagement with music. For the current study, we did not use the Gold-SMI results, but the data are available from the authors upon request.


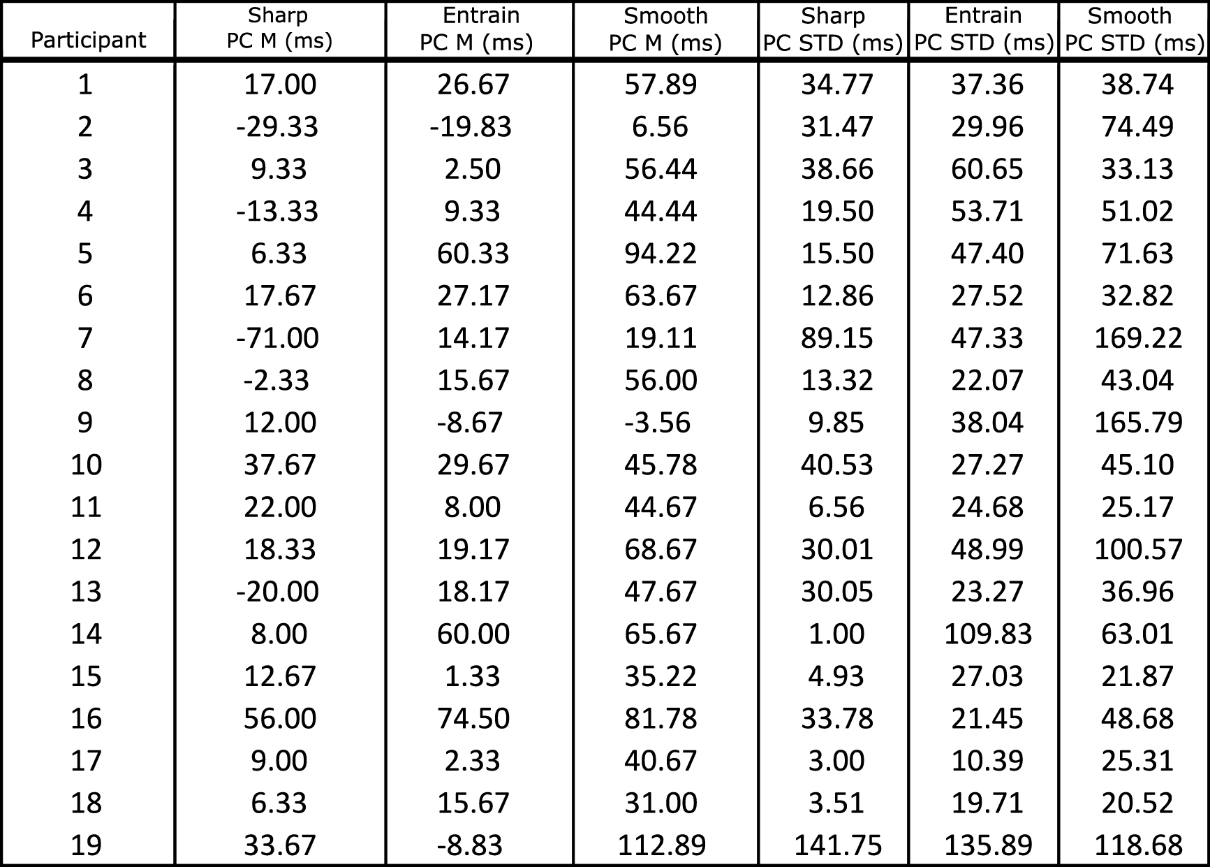


***Supplementary Table 1.*** *Individual P-center locations (PC M: P-Center mean across trials) relative to sound onset and P-center variability (PC STD: P-Center standard deviation across trials) in milliseconds (ms) for all three sound types used in the study.*


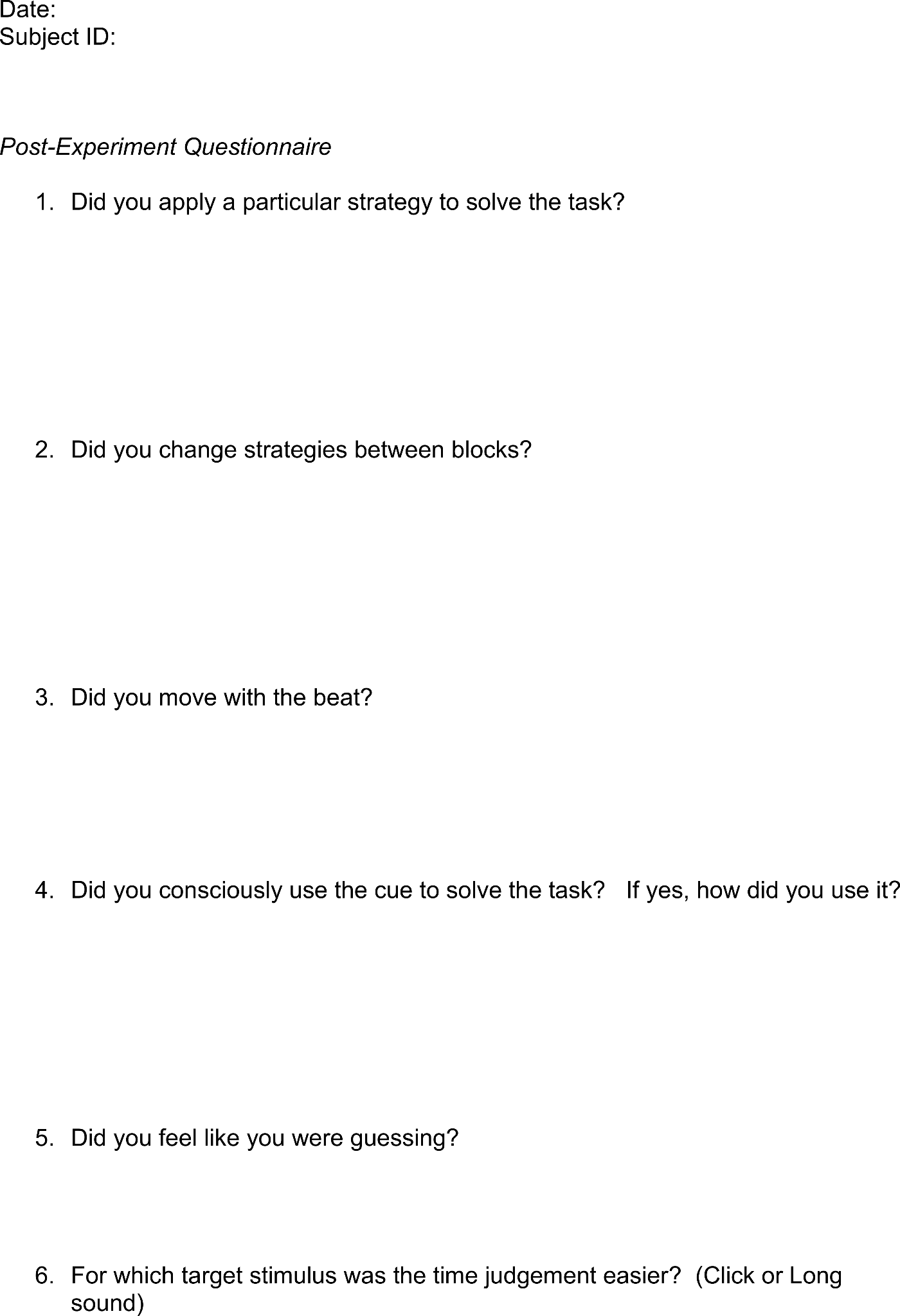


***Supplementary Figure 1****. Post-experiment self-report filled out by the participants.*

**Results of the Post-Experiment Questionnaire**

Participants filled out an open-ended questionnaire to self-report on how they solved the task. In all, 57% of the participants reported that they used a strategy to solve the task, and 31% of the participants used counting as a strategy. In all, 36% of the participants reported changing strategies during the experiment, while 47% did not feel like they were guessing, 32% occasionally felt like they were guessing, and 21% felt like they were indeed guessing with respect to the timing-judgment task. See the main manuscript for the other results of the self-report.


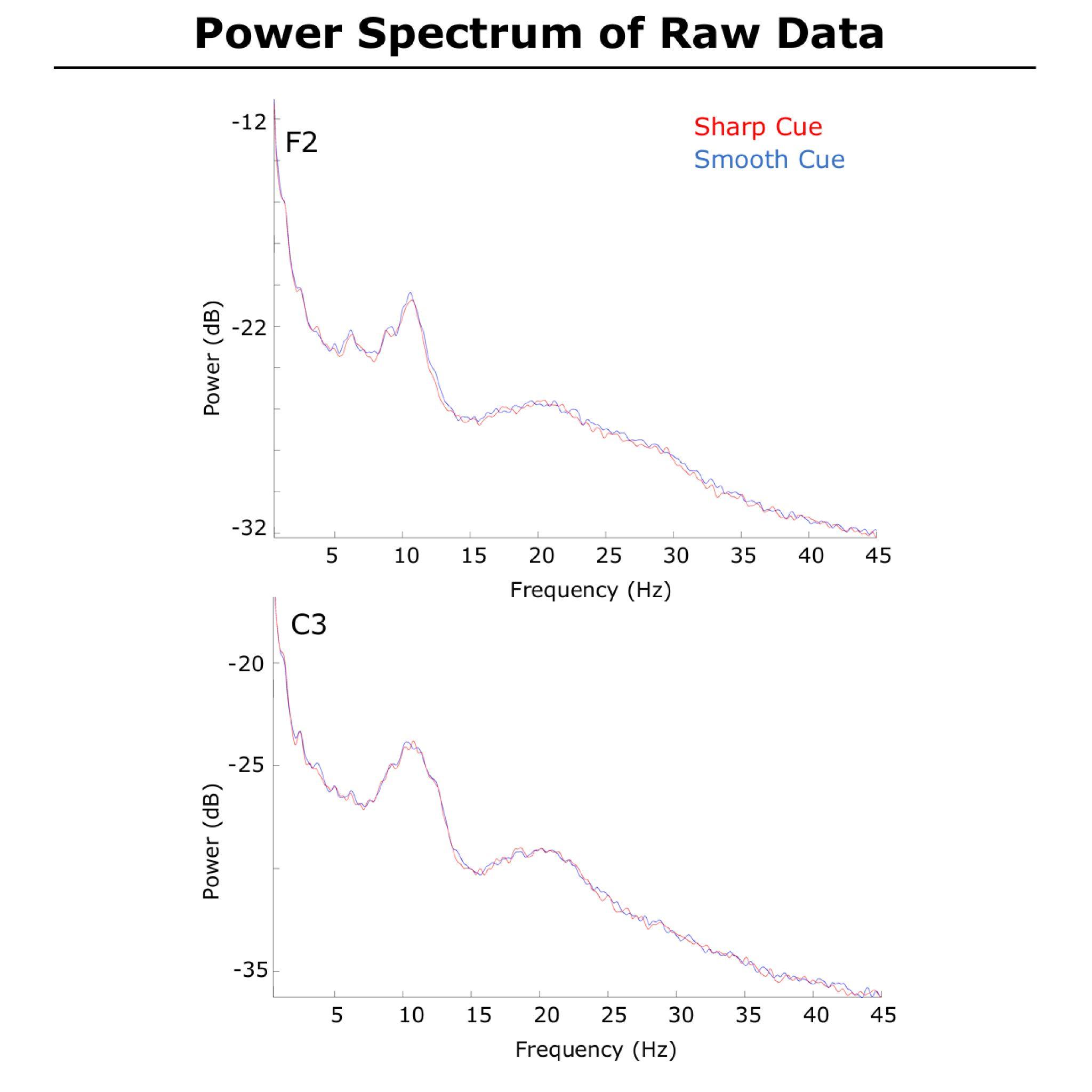


***Supplementary Figure 2.*** *Power spectra for the entire entrainment period for the sharp (red) and smooth (blue) cue conditions for example sensors F2 and C3.*

*
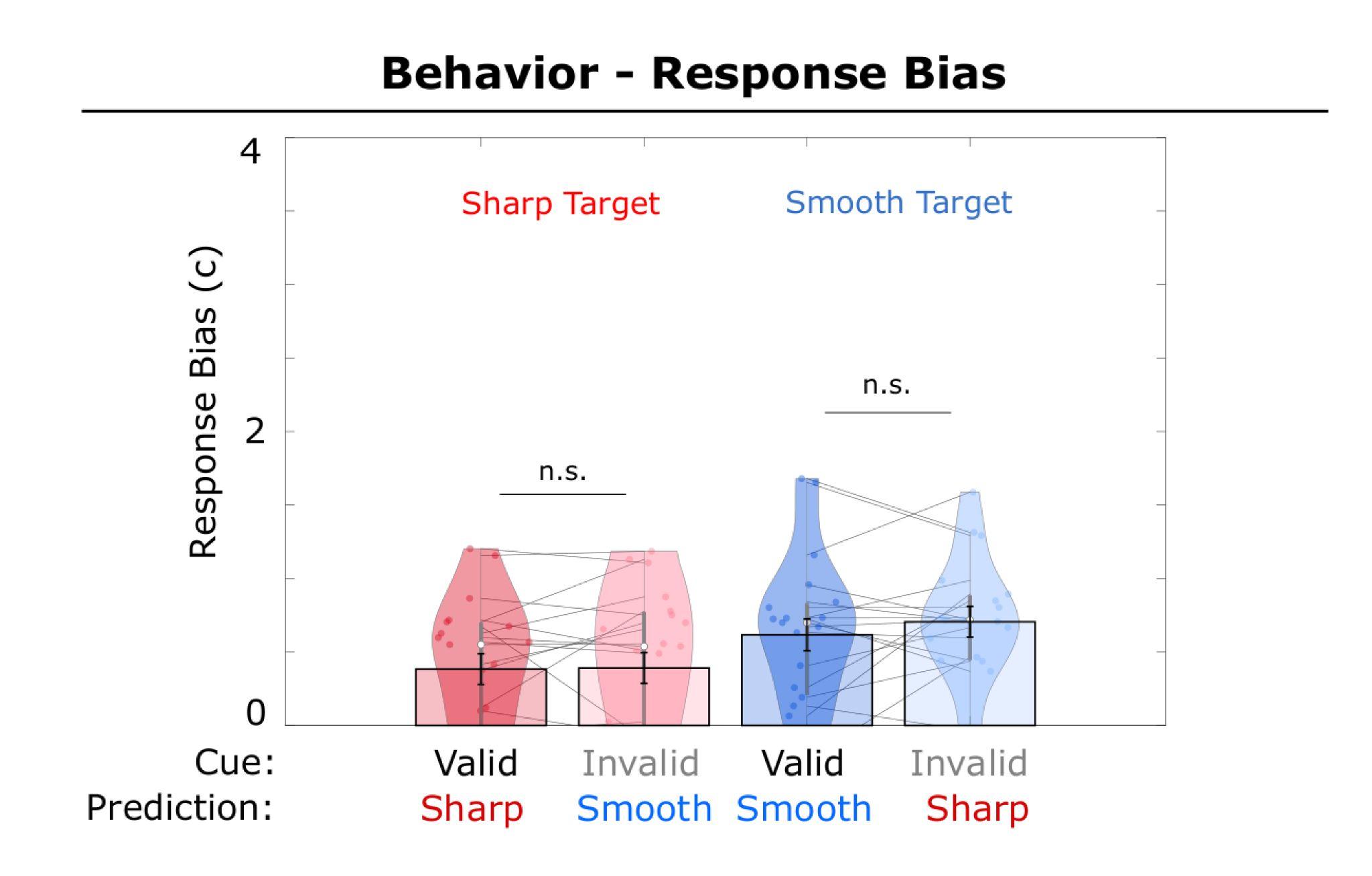
*

***Supplementary Figure 3.*** *Behavioral Results for the timing judgment task.*

*The response bias showed no significant modulations by validity for the sharp (t(18) = -0.09, p = 0.926, two-tailed) or the smooth (t(18) = -1.06, p = 0.302, two-tailed) target condition. Bar plots represent the mean and black error bars represent the adjusted standard error (SE) across participants for a within-subject design, according to the Cousineau-Morey correction ^2^. Violin plots show kernel density estimates and data points. Asterisks refer to significant differences between conditions (*< 0.05, **< 0.01, ***< 0.001). Valid cue conditions are depicted in dark colors, and Invalid cue conditions are depicted in light colors.*


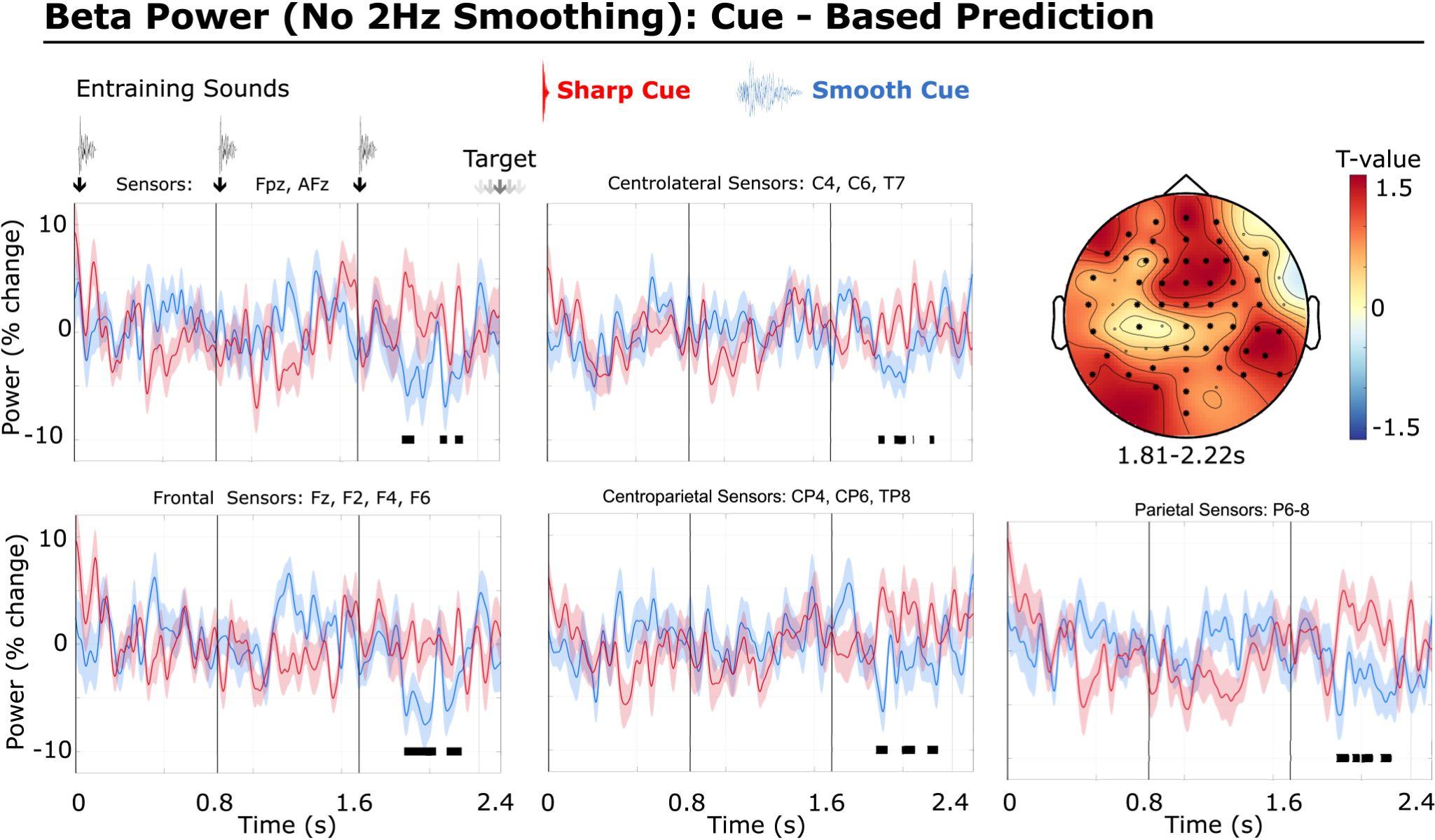


***Supplementary Figure 4.*** *Beta power without smoothing the time series corresponding to a 2 Hz low-pass filter. Significant pre-target beta power enhancement for the sharp (red) versus smooth (blue) target prediction based on the cue (N = 19, cluster T-sum = 2301.65, cluster P-value = 0.007, one-tailed).* *Shaded areas represent the adjusted standard error (SE) for a within-subject design, according to the Cousineau-Morey correction ^2^.* *Black lines indicate significant differences in the temporal dimension. The spatial extent of the significant clusters is depicted by bold stars in the topographical plots. The small gray arrow symbols for the target depict multiple possible onsets for the target according to individual P-Centers.*


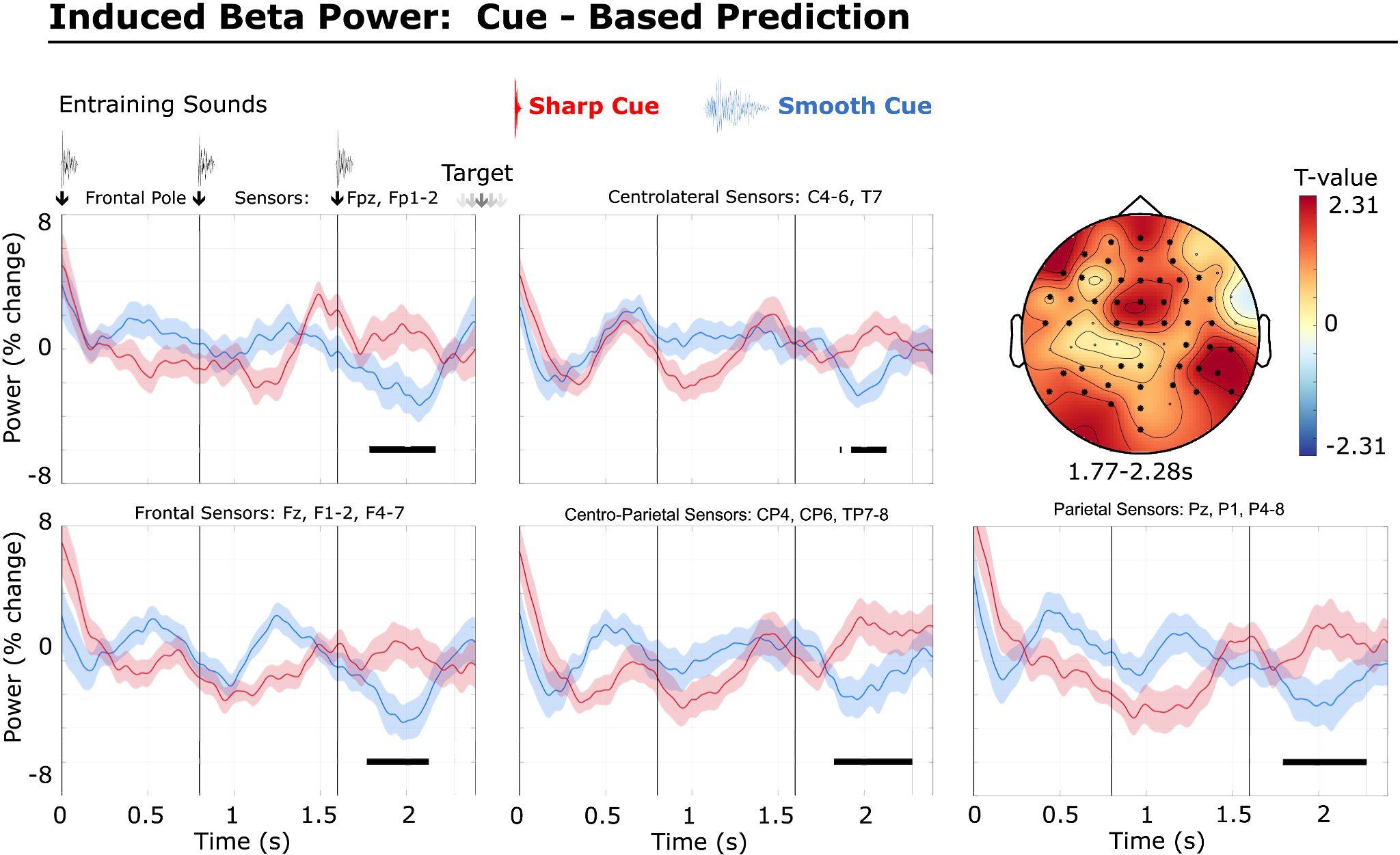


***Supplementary Figure 5.*** *Induced Beta power. Event related potential (evoked response) was subtracted from single trial waveforms to retain induced beta power time series. Significant pre-target beta power enhancement for the sharp (red) versus smooth (blue) target prediction based on the cue (N=19, cluster T-sum = 4543.60, cluster P-value = 0.026, Cohens d = 0.82). Shaded areas represent the adjusted standard error (SE) for a within-subject design, according to the Cousineau-Morey correction ^2^.* *Black lines indicate significant differences in the temporal dimension. The spatial extent of the significant clusters is depicted by bold stars in the topographical plots. The small gray arrow symbols for the target depict multiple possible onsets for the target according to individual P-Centers.*


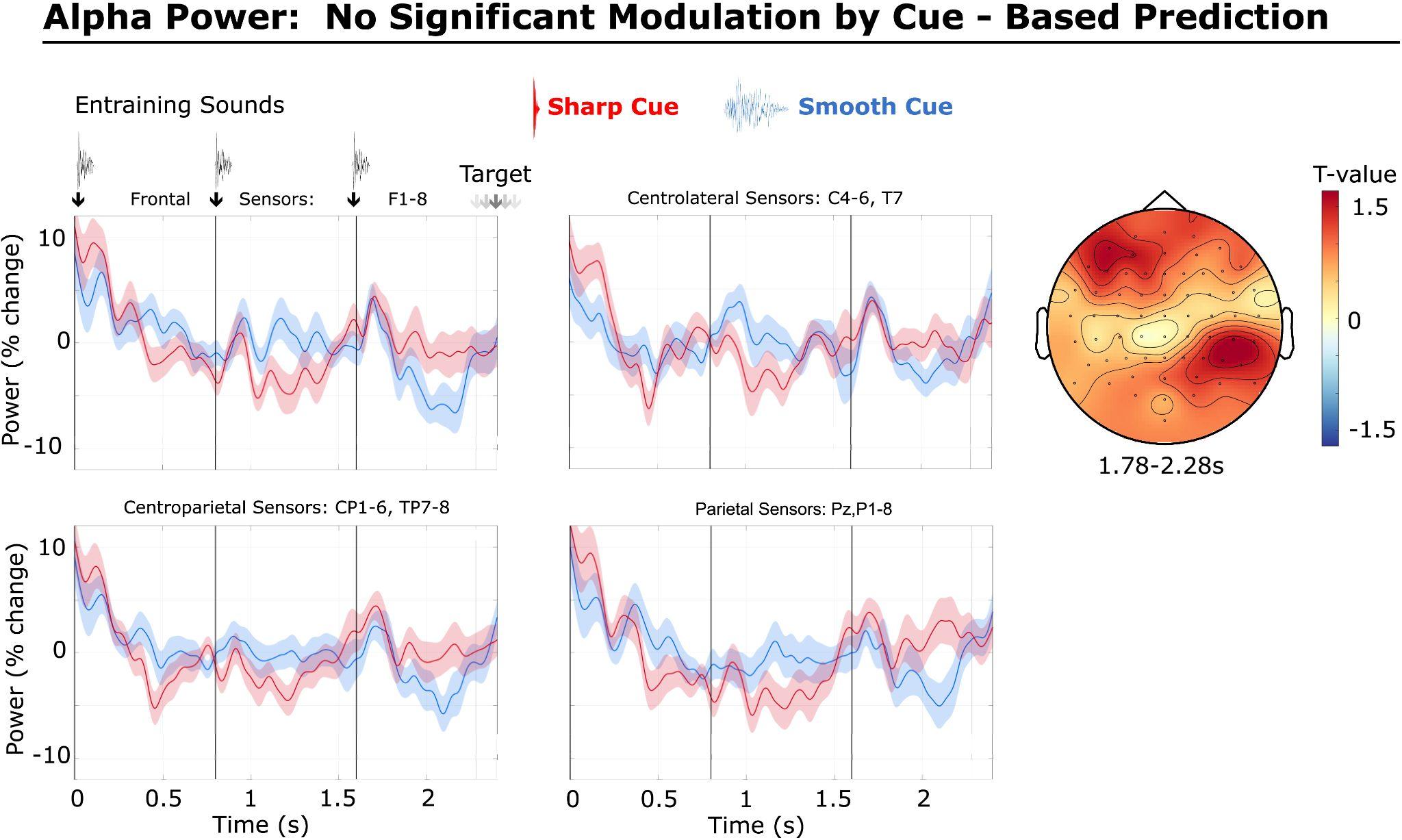


***Supplementary Figure 6.*** *No significant pre-target* *alpha power enhancement for the sharp (red) versus smooth (blue) target prediction based on the cue (N = 19, Cluster T-sum = 6112.44, cluster P-value = 0.116, one-tailed). Shaded areas represent the adjusted standard error (SE) for a within-subject design, according to the Cousineau-Morey correction ^2^.* *The small gray arrow symbols for the target depict multiple possible onsets for the target according to individual P-Centers. There were no significant differences between conditions.*


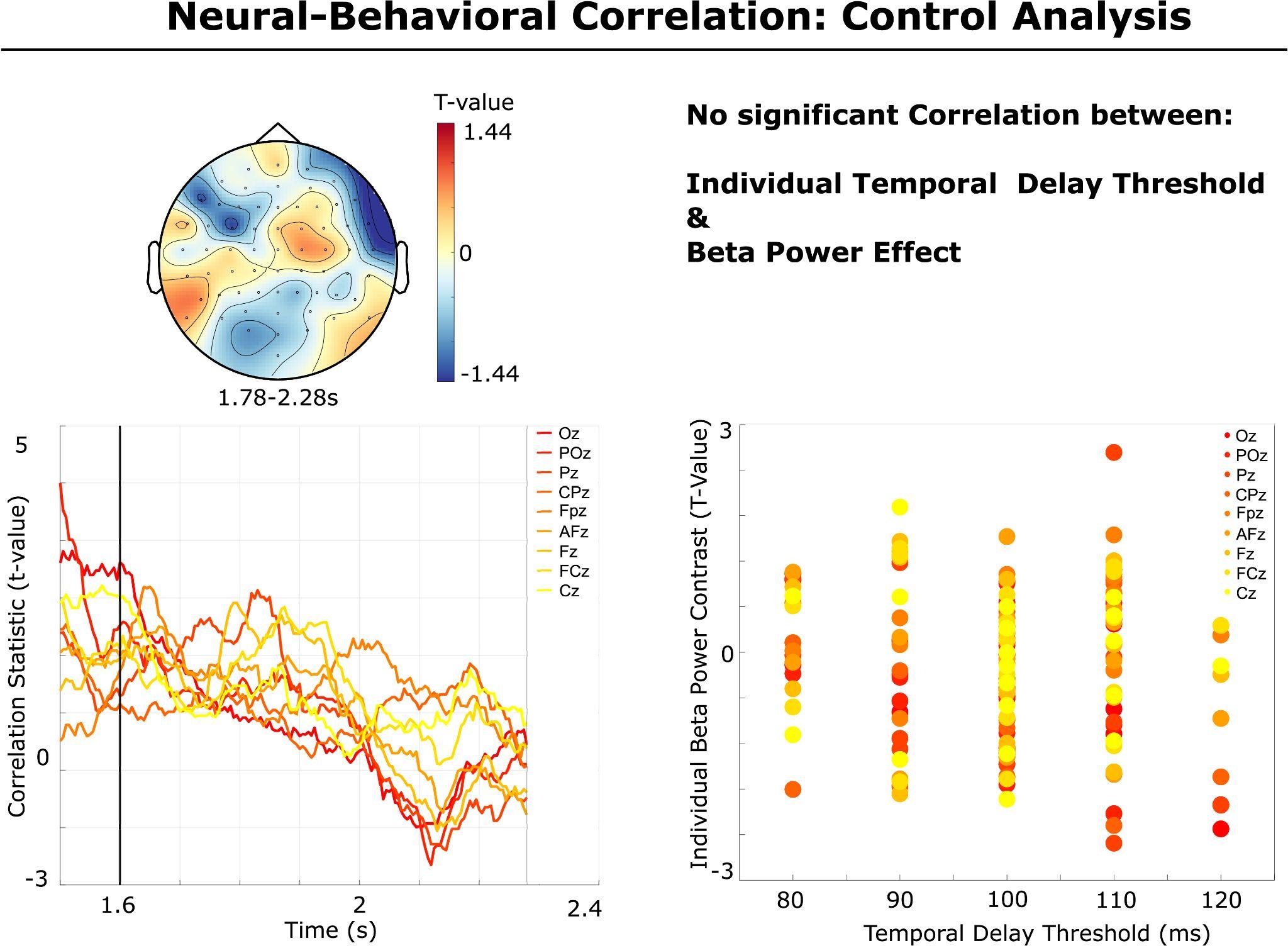


***Supplementary Figure 7.*** *Neural-Behavioral Correlation Analysis to control for a confound due to different difficulty levels (delay thresholds). There is no significant correlation between the individual beta-power effect (t-value) and the individual temporal delay threshold for the sharp target* *(N = 19, positive cluster T-sum = 23.43, positive cluster P-value = 0.717, negative cluster T-sum = -235.95, negative cluster P-value = 0.267).*

*This rules out confounding effects of differences in difficult due to different individual delay threshold values that could have affected the correlation between d-prime measure (performance) and the individual beta power effect (Figure 5 in the main manuscript). Top Left: topographical distribution of the correlation effects. Bottom Left: correlation time courses of midline sensors. Bottom Right: Correlation at the individual subject level (each dot represents a participant for the significant midline sensors).*

**References**

1. Müllensiefen, D., Gingras, B., Musil, J. & Stewart, L. The Musicality of Non-Musicians: An Index for Assessing Musical Sophistication in the General Population. *PLOS ONE* **9**, e89642 (2014).
2. O'Brian, F. & Cousineau, D. Representing error bars in within-subject designs in typical software packages. The quantitative methods for pyschology 10.1, 56-67 (2014).
